# Development of the Niggle App for Supporting Young People on Their Dynamic Journey to Well-being: Co-design and Qualitative Research Study

**DOI:** 10.2196/21085

**Published:** 2021-04-20

**Authors:** Stoyan R Stoyanov, Oksana Zelenko, Aleksandra Staneva, David J Kavanagh, Calvin Smith, Gavin Sade, Jessica Cheers, Leanne Hides

**Affiliations:** 1 QUT Design Lab Creative Industries Faculty Queensland University of Technology Brisbane Australia; 2 Division of Advocacy and Research yourtown Brisbane Australia; 3 HORÓ Counseling Centre for Women Brisbane Australia; 4 Centre for Children's Health Research School of Psychology and Counselling Queensland University of Technology Brisbane Australia; 5 Office of Medical Education Faculty of Medicine The University of Queensland Brisbane Australia; 6 Creative Industries Faculty Queensland University of Technology Brisbane Australia; 7 School of Psychology Faculty of Health and Behavioural Sciences The University of Queensland Brisbane Australia

**Keywords:** mHealth, adolescence, youth, young people, well-being, co-design, participatory design, qualitative research, thematic analysis, recovery, visual methods

## Abstract

**Background:**

Adolescence is a life stage characterized by intense development and increased vulnerability. Yet, young people rarely seek help for mental health, often due to stigma and embarrassment. Alarmingly, even those who do seek help may not be able to receive it. Interventions focused on well-being offer a protective factor against adversity. Highly effective, innovative, theoretically sound, accessible, and engaging mobile health (mHealth) interventions that can be used to look beyond mental ill-health and toward mental well-being are urgently needed.

**Objective:**

We aimed to explore how young Australians conceptualize and construct recovery journeys from feeling unwell to being well in order to inform the conceptual design of a youth-led information-, resource-, and support-focused mHealth intervention.

**Methods:**

A sample of young people, grouped by age (12-15 years, 16-19 years, and 20-25 years), took part in 3 in-person participatory design workshops (per group). Young people’s understanding and representation of well-being, feeling unwell, and the recovery journey were investigated using visual and linguistic data collection methods: photo elicitation and journey mapping. A social constructionist perspective was used for thematic analysis to produce a conceptual model of the recovery journey. A mobile app was co-designed and all app functions were mapped through iterative development and testing by young people and a team of psychology, research, design and information technology experts.

**Results:**

Young people (n=25) described a 6-stage journey with specific barriers and coping strategies. The findings, when situated within the personal recovery framework in mental health, emphasize the cyclic and iterative model of change. Through co-design, the new app—Niggle—was conceptualized as a visual representation of an amorphous problem, which can be addressed through app functions corresponding to the most helpful strategies that young people used to progress through the stages of their recovery journey.

**Conclusions:**

Niggle is available to offer support to young people for a range of problems and provides a hot link to counseling services in Australia. This paper elaborates on the process of in-depth qualitative data collection through visual, linguistic, and co-design methods. The findings of this study give insight into young people’s understanding of well-being and recovery. This paper could aid the development of high-quality personalized mHealth interventions and support resources.

## Introduction

### Young People and Mental Health

Adolescence is characterized by intense cognitive, emotional, social, and physiological growth; increased vulnerability [1,2]; and deficits in emotion regulation capability [3-6]. Young people (under the age of 25 years) experience high levels of mental disorders [1,7] and increased exposure to risk factors [8,9]. In recent years, however, there has been a shift of focus in psychology from mental illness and pathologizing ill health toward positive psychology approaches, well-being, recovery-oriented approaches, and resilience- and strength-based frameworks, which is exemplified in research on well-being.

### Well-being

Commonly described as the experience of positive emotions about one’s life, such as happiness, life satisfaction, and positive functioning, well-being is illustrated by a sense of fulfilment and engagement in life [10]. In 1998, Keyes [11] proposed the dual-continua mental health model which posits that mental illness and mental well-being lie on two separate but related continua, such that mental well-being cannot be merely defined as the absence of mental illness. Further research suggested that mental illness and well-being represent two distinct subcomponents of an overarching construct of mental health [12]. Well-being recovery models describe the transcendence of symptoms into a renewed sense of meaningful life, despite the limitations of mental illness [13]. Greater well-being is associated with fewer symptoms of mental illness and reduced incidences of behavioral issues such as criminality, substance use [14,15], and physical illness [16]. Thus, recent approaches to mental health propose a shift from the treatment and prevention of mental illness toward the enhancement of well-being [17].

### Lack of Well-being in Young Australians

Keyes [18] defines *flourishing* as the presence of emotional, psychological, and social well-being. The lack of well-being, defined as *languishing*, is characterized by low levels of these characteristics. Young Australians consistently report lower levels of well-being than adults [9,19-22]. According to these definitions, lower youth well-being levels reflect emotional, psychological, social, and help-seeking–related factors. (1) Emotional—Young adulthood is a developmental period frequently associated with an increased exposure to risk factors including increased emotional imbalance, developing emotion regulation skills, and the stressful period of transition to adulthood, placing demands on coping resources [8,9]. Young people experience increasing emotional unrest and have low capacity to effectively regulate emotions [4,5]. (2) Psychological—Most mental illness emerges before the age of 25 years [7]. In Australia, mental disorders commonly affect children and adolescents (aged 4 to 17 years), with 13.9% reporting a mental disorder in the past 12 months [23]. The 2019 National Health Survey in Australia [24] revealed that young people aged 15 to 24 years experience the highest levels of mental or behavioral conditions compared to all other age groups. (3) Social—Relationships play a central role in the well-being of young people [25]. The most common reason for individuals aged 12 to 25 years contacting major Australian youth counseling services such as Headspace or Kids Helpline was “relationship problems,” which includes family, partner, or peer relationships [26,27]. (4) Help Seeking—It is concerning that only one-third of young people with mental disorders seek help. This is due to barriers such as stigma, confidentiality issues, lack of accessibility, self-reliance, lack of knowledge about mental health services, and fear or stress about the act of help seeking or the help source itself [28-31]. Alarmingly, even those who seek help may struggle to receive it. For example, Kids Helpline, Australia’s largest telephone and web-counseling service, is unable to meet demand, with continuously decreasing yearly response rates of approximately 50% [32,33]. Highly effective, innovative, accessible, and engaging support solutions that promote well-being are urgently needed [29].

### The Promise of Mobile Health Technology

The ubiquity and increasing functionality of modern mobile health (mHealth) technology offers promise for filling the gap by addressing barriers, such as stigma, confidentiality concerns, or difficulty of accessing support, to help seeking [34]. Young people are among the highest adopters of innovative technologies [34]. This presents an excellent opportunity for the development of novel, accessible mHealth interventions that offer reliable information and support.

Quality mHealth interventions are engaging, functional, professionally designed, and contain reliable information [35,36]. Several factors are essential to increase an intervention's potential for uptake, use and efficacy. This includes involving stakeholders in co-design, through participatory design workshops; conducting in-depth research of the phenomena which the mHealth intervention is designed to address [12,37,38]; involving researchers and design experts to interpret and translate participatory design workshop data into the design of the intervention; and involving health experts to assess its alignment with established theoretical models [39].

The purpose of this project was to qualitatively explore how young people conceptualize and construct individual recovery journeys, from being unwell toward experiencing greater levels of well-being. The findings were used to inform the conceptual design of a youth-led information-, resource-, and support-focused mHealth intervention for the largest support service for young people in Australia (Kids Helpline).

## Methods

### Participant Recruitment

Ethics approval from Queensland University of Technology was granted for all stages of this project (Human Research Ethics Approval Number 1600000956). Recruitment was done via Facebook and researcher networks within Brisbane, Australia. Young people were invited to attend a series of workshops with the aim of co-designing a new digital tool for youth well-being. Quota sampling was applied and included age (12-25 years) and English language as participation criteria. Information sheets were sent to 35 young people who contacted the team. We grouped participants by age (3 separate age groups were formed—12-15 years; 16-19 years, and 20-25 years) to ensure variability of age-specific experiences and to reduce age-related peer pressure within each group. Consent (including parental consent for the youngest cohort) was obtained before participation. A series of participatory design workshops (3 per age group) were conducted between February and March 2017; each workshop lasted approximately 2 hours each. Participants were offered $30 (approximately US $23.25) or a movie voucher for their time.

### Research Design

The participatory design workshops [38,40,41] combined visual and linguistic data collection methods—photo elicitation [42,43] and journey mapping—drawn from user experience design [44,45], to ensure a rich variety of concepts. The use of linguistic methods alone creates limitations, especially for younger participants [42,46-48]; therefore, the young people were provided with multiple communication modalities to explore individual experiences and understanding [45,49,50] of well-being, being unwell, the recovery journey between, and potential technological solutions. 

### Procedure and Analysis

#### Step 1

To immerse participants in the topic of well-being without priming them with researchers’ definitions, we first asked them to create lists of associations for the terms *well-being* and *being unwell*.

#### Step 2

Each participant was offered a set of 126 images cut out from different sources (Multimedia Appendix 1), which included a large variety of colorful or black-and-white images, patterns, textures, and icons that were representative of relationships, emotions, nature, activities, popular app icons, and abstract images. The images in the set were aggregated and refined by the research team and a group of youth consultants. Participants were invited to (1) select images associated with well-being, (2) select images associated with being unwell, and (3) create separate collages for each category.

#### Step 3

Participants drew, on a blank sheet, a *recovery journey* depicting a hypothetical or experienced journey from being unwell to well and were provided with a specially designed selection of lines and arrows to mark the steps in meaningful ways (ie, indicate difficult steps, easy steps, barriers, etc). The team deemed it ethically appropriate to phrase journeys as hypothetical scenarios, to allow participants the flexibility to impersonally reveal potentially distressing, confronting, and stigmatizing personal experiences in front of the group. Participants engaged in continuous verbal annotations of their own maps, which was followed by a group synthesis of all journeys.

#### Step 4

**Figure 1:**
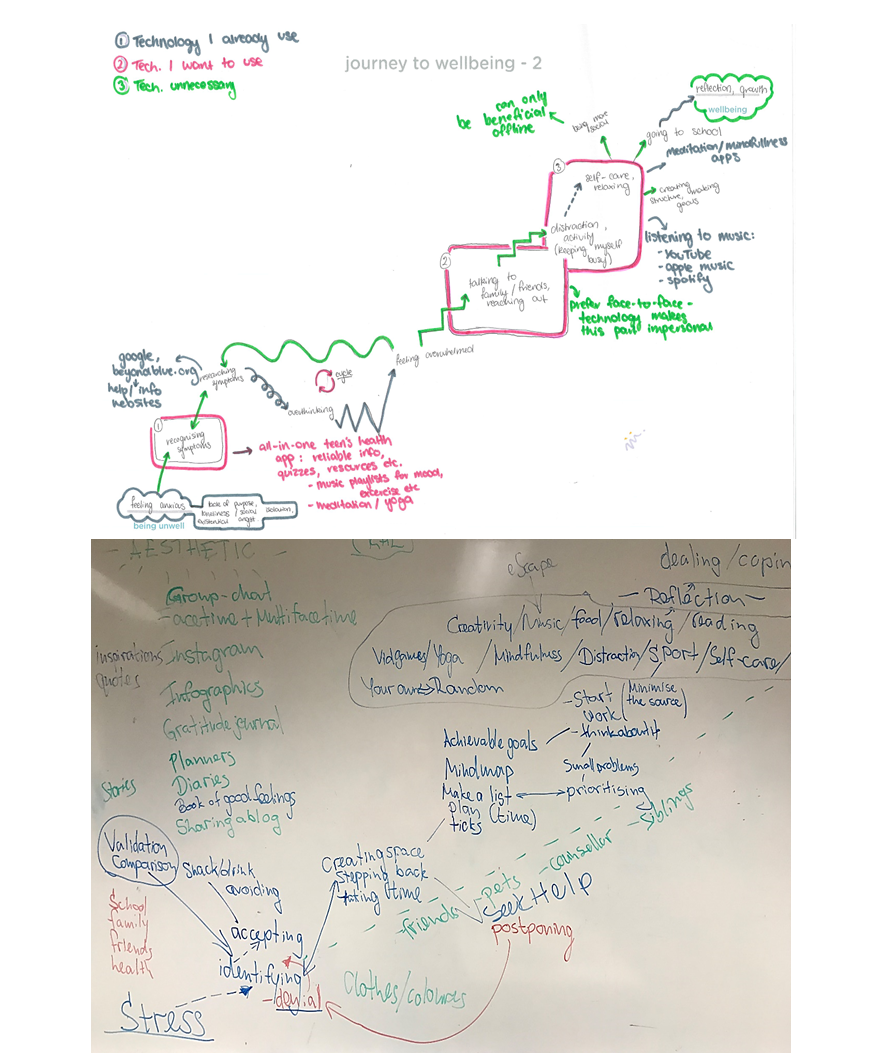
Individual and group journey maps.

Participants were asked to reflect on their journey maps and consider which steps could be assisted by technology and in what capacity (Figure 1). Thereafter, in-depth group discussions aimed to answer, “What type of technology would be best suited to provide assistance?” and “What technological functions would best address youth needs in progressing through the journey to well-being?” Participants were invited to work in pairs to create their most-desired mHealth tool (ie, with no limit to imagination and if resources were unlimited) to support the journey to well-being. Decisions to end data collection were based on saturation of emerging themes (Multimedia Appendix 2).

### Data Analysis

Analysis was largely informed by a social constructionist perspective [51], which assumes that people construct their own meaning of reality through interactions with others within a social space. This theoretical framework also allows for the interpretation of both linguistic and visual methods [49]. Hence, we viewed the data as forms of self-accounting in which participants were attentive to the expectations associated with the production and reception of identities, both in immediate and broad social contexts.

Verbal data were transcribed verbatim and COREQ (Consolidated Criteria for Reporting Qualitative Research) [52] guidelines were followed. Codes were organized into thematic groups following inductive thematic analysis [53]. One team member with 7 years of experience conducting qualitative psychological research coded the transcripts in 3 iterative steps. After each coding step, a discussion was held with 2 additional team members, who were also experienced in qualitative research, to reach agreement on the codes, themes, and theme names. Visual data were interpreted using photo elicitation analysis [42] and image clustering patterns were mapped over the verbal elaborations of the thematic analysis [53]. Comprehensive notes (on both verbal and visual data) were taken during the iterative process and incorporated in the final analysis.

### Intervention Design and Development

Aggregate technological solutions data were used to develop the concept design, storyboard, and functions of the mHealth tool, unanimously conceptualized by participants as a mobile app. The functions, information, and interface of the app were designed using iterative co-design—with young people and an expert development team. Principles outlined by the Mobile App Rating Scale (MARS) [35], which outline a set of quality criteria and consist of 29 items organized in 4 objective (engagement, functionality, aesthetics, and information quality) and 2 subjective (subjective quality and perceived impact) subscales, were followed. MARS provides a checklist of criteria and definitions to assist developers in creating high-quality health apps.

### The Role of the Researchers

The team consisted of researchers, designers, information technology developers, and young people. The collaborative approach served to ensure the thorough, interdisciplinary exploration of the psychological phenomena and the appropriate, grounded in theory, and participant data co-design, and development of the new technology.

## Results

### Participant Characteristics

A total of 25 participants residing in an urban setting in Australia took part in the study (Table 1). We planned to have each group be a mixed of genders (all participant identified as either male or female); however, 3 male participants who signed up for the 12-to-15-years-old age group did not attend on the day and did not respond to further communication attempts. Thus, this age group was only represented by female participants. Two 16-to-19-years-old groups were run in parallel because of a large amount of participant interest in that age range.

**Table 1:** Participant characteristics.

Characteristics			Age group				
		12 to 15 years (n=7), n		16 to 19 years (n=14), n		20 to 25 years (n=4), n	
**Gender**							
	Female	7		8		3	
	Male	0		6		1	
**Ethnicity**							
	White, Australian	6		6		3	
	White, New Zealander	0		1		0	
	Asian	0		5		1	
	Middle Eastern	1		1		0	
	European	0		1		0	
**Education**							
	Completed primary school	0		1		0	
	Partially completed high school	7		9		0	
	Completed year 12	0		4		2	
	Bachelor's degree	0		0		2	
**Employment status**							
	Full-time employment	0		0		0	
	Part-time or casual employment	0		4		1	
	Full-time student	7		4		2	
	Part-time student	0		1		0	
	Unemployed	0		5		1	
**Living arrangement**							
	With parents or guardians	7		12		2	
	With friends or in shared accommodations	0		2		1	
	Alone	0		0		1	
**Relationship status**							
	Single	7		13		3	
	In an exclusive relationship	0		1		1	
**Currently receiving psychological treatment**							
	No	6		13		2	
	Yes	1		1		1	
	Prefer not to say	0		0		1	

### Themes

#### Overview

**Figure 2:**
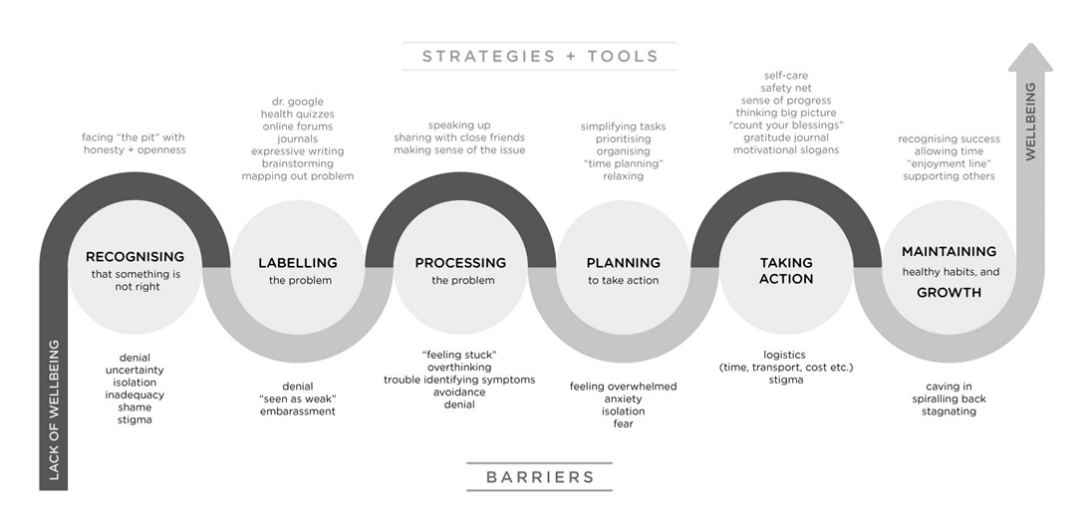
Young people’s journey toward well-being.

Young people indicated that the recovery journey was experienced as a complex, multifaceted phenomenon. Thematic analysis suggested that the journey consisted of 6 separate, dynamically related stages (Figure 2). It commonly started as “depression,” “sadness,” “loneliness and isolation,” and “feeling fearful”, then progressed toward “feeling happy, loved, and accepted” or “overcoming fear and sadness.” The described journey stages significantly overlapped across all age groups and are presented here as forming a sequence, however, the journey could be nonlinear.

#### Stage 1: Recognizing That Something Is Not Right

This first stage was described as

the pit…it’s like a learning curve. So you get so deep into it and you don’t really know how to climb back out. So it’s like you’re kind of like you want to recognise the problem but you don’t want to recognise those steps cause you’re scared or anxious.

Participant, 16-to-19-years-old age group

You have to accept that something is wrong. I think a big part of it is you know something is wrong but being like “okay I need to get help for this. I can’t do it on my own.”

Participant, 16-to-19-years-old age group

Barriers that were described included a sense of uncertainty, insecurity, and denial that lead to isolation and loneliness. Participants recognized that feelings of depression or anxiety could result in experiencing shame, inadequacy, and stigma, and if left unacknowledged, such emotions may lead to further stagnation. Conversely, we gathered that facing the pit was a positive first step of accepting such feelings with honesty and openness to overcome “the negative spiral down.”

I feel like you just try and push it away and ignore it.... That’ll stress me out even more.

Participant, 12-to-15-years-old age group

**Figure 3:**
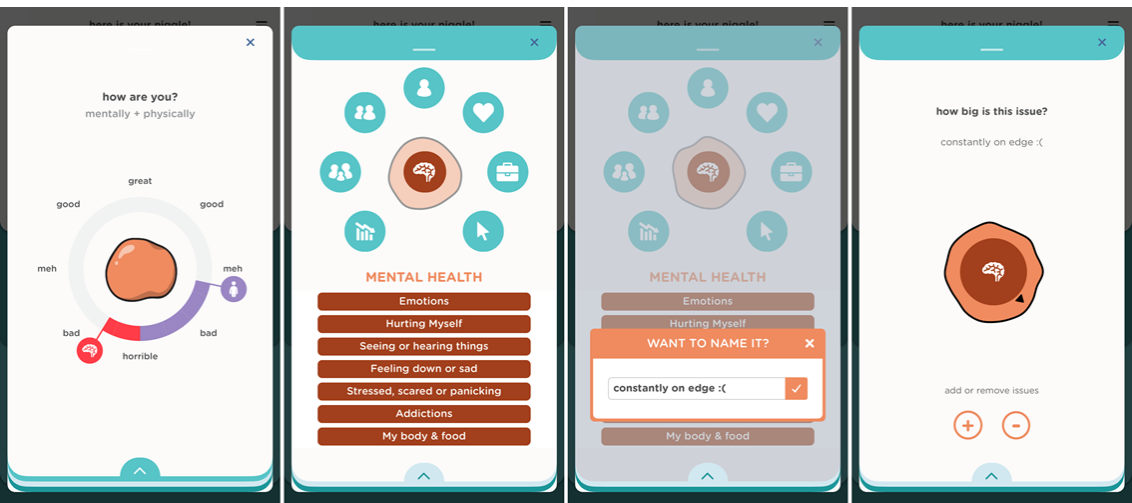
Well-being log, labeling, and resizing the Niggle.

Participants suggested that the app should contain tools allowing reflection on the current physical and psychological state and a selection of common problems for their age to support this step of the recovery journey. The design solution included sliders used to modify the color of the amorphous problem, which the young people called “niggle,” and a selection of issues from Kids Helpline’s youth problem classification lists (Figure 3).

#### Stage 2: Labeling the Problem

Young people could move onto accepting their niggle to get closer to overcoming it by recognizing it as a problem and giving it a name. Participants indicated that this stage was difficult due to the barriers of denial or avoidance.

...you can still identify it and not want to do anything about it because you either don’t have the energy to look into it, or you don’t have the self-esteem to think you deserve better.

Participant, 16-to-19-years-old age group

Participants described useful strategies such as researching symptoms online, looking up health quizzes, or accessing online forums. They recognized that checking symptoms on Google was risky due to the lack of distinction between helpful and hazardous advice. Fleshing out the problem and giving it a name represented a greater sense of control, a broader perspective, better self-awareness, and acceptance. Participants discussed that acknowledging embarrassment, shyness, and nervousness at this stage was common and a key strategy used by participants to normalize their experience and continue the journey.

That’s pretty hard, like you would have to really sit down with yourself and say “alright, I don’t feel comfortable doing this, but I think I might have to.” You could look online and see ways you could like overcome pride... Yeah you have to put effort into identifying it means you want to do something about it which means you care about yourself.

Participant, 12-to-15-years-old age group

Successful tools, described by participants, for recognizing and labeling an issue included journaling, expressive writing, brainstorming, and mapping out the problem.

Sometimes I just write down how I feel and like what’s going through my mind and I go “what could this be?”

Participant, 12-to-15-years-old age group

To support this stage of the journey, the app depicts the problem as an amorphous niggle slowly taking shape. Users can choose up to 3 issues, give them their own labels, and adjust the size of each according to its gravity (Figure 3).

#### Stage 3: Processing the Problem

The next stage of the journey toward well-being involved a contemplation period of stepping back and creating space in order to process the confrontational experience of the realization that “something’s not right” [Participant, 16-to-19-years-old age group]. This stage required persistence, deliberate reflection, and the ability to stay with the uncomfortable emotions. Young people spoke of prioritizing, rationalizing, and separating oneself from the problem in order to process it. They described recognition as being slow, “shaky,” and nonlinear. A participant compared it to the “learning of a new complex skill, such as coding,” which requires time and diligence and builds upon layers of newly acquired confidence.

Wellbeing and having strong mental health is another skill you have to gradually build. Learning how to program websites, I’ve always had to refer back to all the different codes but now...I feel confident I have those codes...Once you get into those healthier habits...physically or intellectually you’re more independent, your wellbeing will be better, and you will be able to move through the process.

Participant, 20-to-25-years-old age group

Barriers mentioned by the participants included feeling stuck, difficulties identifying the symptoms, overthinking, avoidance, or rushing for “quick fixes.” To overcome these, participants relied on speaking up, educating themselves, sharing with close friends, and comparing to others with similar experiences, but not yet focusing on solutions.

Yeah, if it's a problem that you can't change, then stepping back is probably a good thing, but if it's a problem that you can change...

Participant, 12-15-years-old age group

...stepping back I think has to do with like, identifying and then being able to think about priorities.

Participant, 12-to-15-years-old age group

Yeah like um, separating yourself from an issue.

Participant, 12-to-15-years-old age group

**Figure 4:**
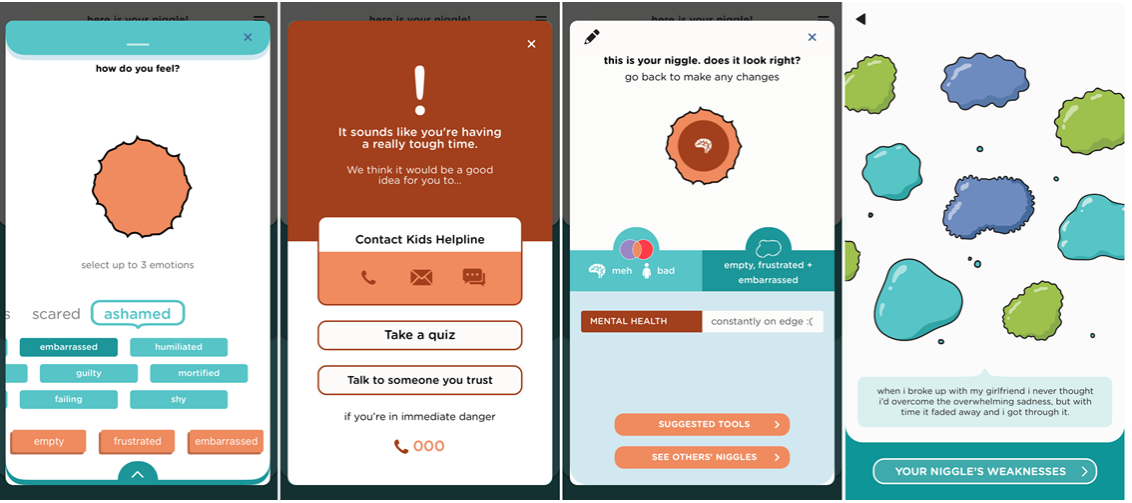
Processing screens.

To support progress through this stage, the app offers 2 elements. (1) The app invites users to take a moment to reflect on their emotions; they can select up to 3 emotions to associate with their current niggle, which changes its shape on the screen. (2) Once this 3-stage setup process is complete, it presents other users’ niggles—a scrollable screen full of amorphous shapes with different colors and outlines. Tapping on any one of the shapes opens a support message from other users who have dealt with similar niggles (Figure 4).

#### Stage 4: Planning Action

Participants shared that actively approaching a problem, learning more about it, brainstorming solutions and planning appropriate steps followed in the recovery journey once the processing stage was successfully completed and enough strength was gained. Planning involved setting achievable goals and a to-do list. Simplifying tasks by breaking them down into small steps, prioritizing and organizing a predictable timeline rather than dwelling on the big picture helped significantly. Rewards and “tick boxes” were regarded as positive reinforcement for sticking with the plan.

When I'm overwhelmed I know that I like to make a list of my priorities and like, things that I need to do just to kind of like, see it and tick it off...

Participant, 12-to-15-years-old age group

Yeah like kind of breaking down everything you need to do into smaller bits and like kind of, working through it slowly.

Participant 2, 12-to-15-years-old age group

Making time for “chilling or meditating,” listening to music, or engaging in pleasant hobbies were described by participants as additional tools in the arsenal of coping strategies. They mentioned barriers that included feeling overwhelmed by tasks, fear, increased anxiety, and emotional isolation. Some participants shared that planning could be a challenging and confronting experience, as it requires a greater realization of one’s concerns and the difficulty of overcoming them. Others mentioned their worries regarding help seeking such as distrust of the sources of support.

**Figure 5:**
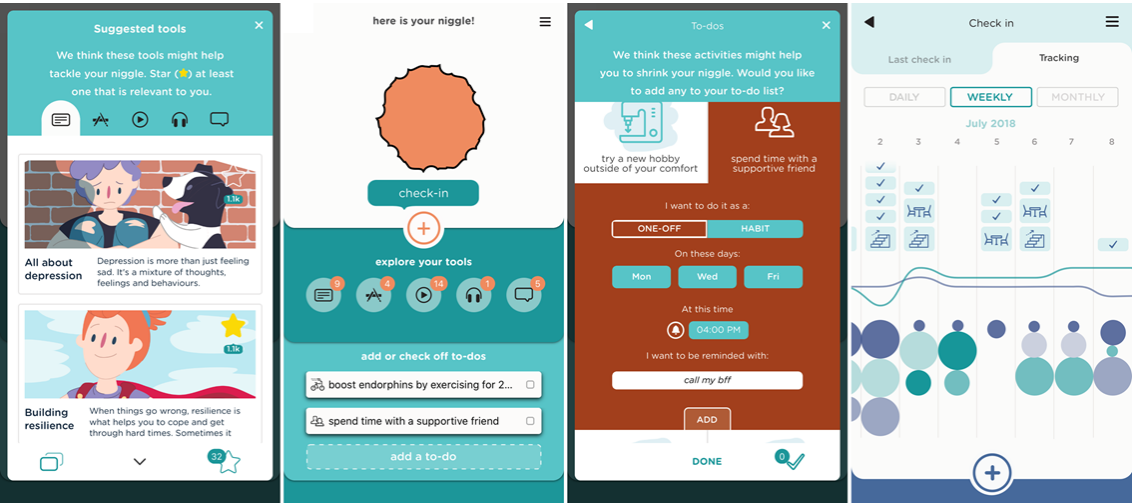
Learning and Planning screens.

As learning and decision making characterized this step, the new app incorporates 2 key functions. (1) Users are presented with carefully researched or specially developed information about the selected issues in the form of tip sheets, videos, podcasts, relevant apps, and recovery stories from peers. Since searching the web for information can be problematic, resources were selected by a team of psychology students and reviewed by experts. (2) Young people are offered a list of helpful activities (to-do lists) specific to their niggle, which were developed by youth counselors. These activities can be scheduled directly into the app’s integrated calendar (Figure 5).

#### Stage 5: Taking Action

This stage was characterized as actively taking steps to resolve the problem. Young people felt particularly vulnerable at this stage of recovery. The intimidation of taking the first step, lack of accessibility to support, time limitations, and practical issues such as transportation and the cost of psychotherapy were all mentioned; however, participants indicated dealing with public and perceived stigma and shame (for speaking up about mental illness) was the most prevailing problem. Thus, escapism and quick fixes (such as unhealthy eating; binging on alcohol, drugs, shopping, or video games) were described as tempting alternatives.

I think of escaping, it helps for a part. It helps the issues dull down and it helps you accept the issue and kind of deal with it. But after you escape you have to deal with the emotion.

Participant, 20-to-25-years-old age group

**Figure 6:**
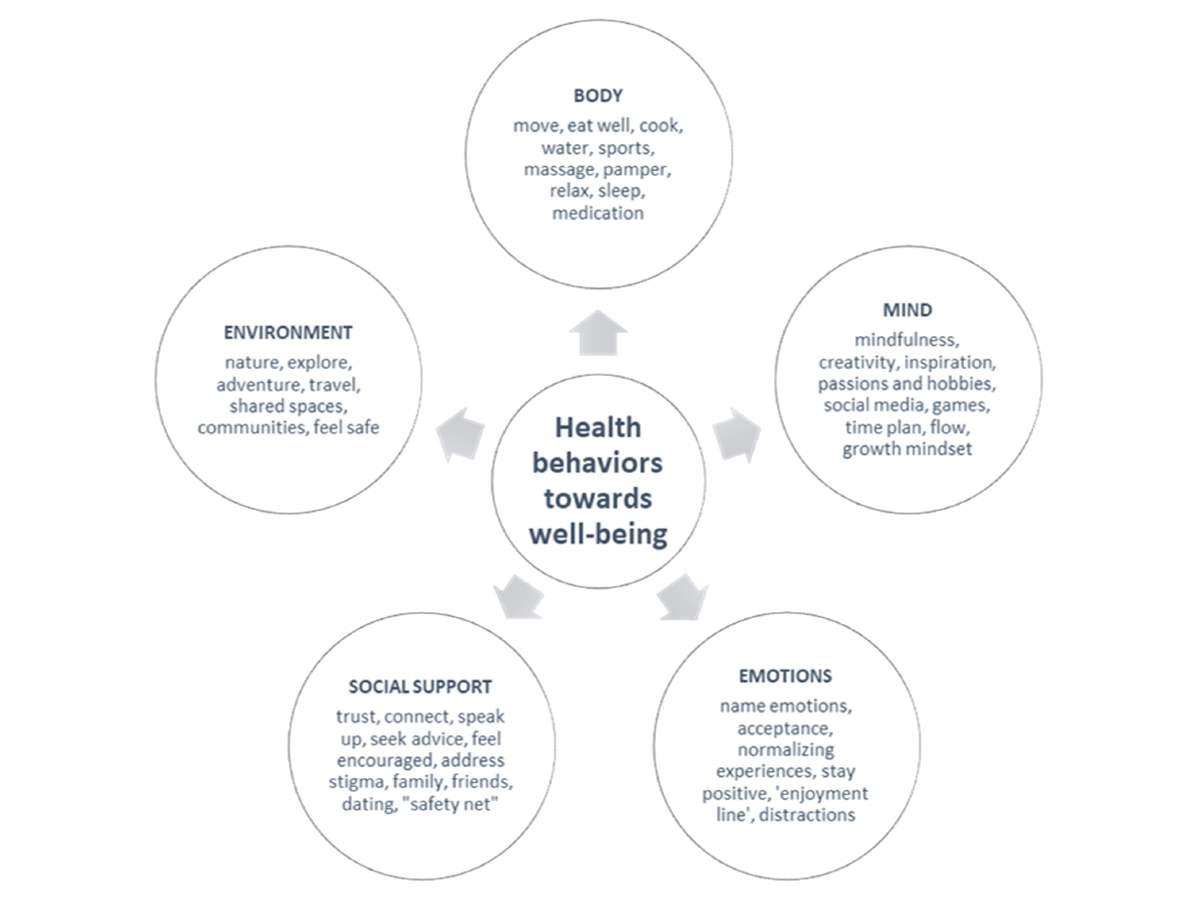
Health-enhancing behaviors for well-being.

Helpful behaviors included speaking with family, friends, psychologists, counsellors, online or telephone support services or community groups (Figure 6). Technology was found to be both useful (online counseling, forums, playing video games, or using apps and websites) and unhelpful, particularly, the use of social media.

...stay present and truly with friends rather than on phones...

Participant, 16-to-19-years-old age group

Focus on stuff that you were able to do; and you feel proud of yourself... And from there, I put the gradual steps; to finding someone to talk about it... Because once you know that your feelings are valid, there’s a part of your mind that wouldn't know what to do and that you might need help.... And then meanwhile, you can learn new things or find new hobbies that will occupy you.

Participant, 20-to-25-years-old age group

Positive relationships and trustworthy communities served as a powerful “safety net to catch you if you were to fall back into bad habits.”

It’s not like it’s a solo mission. You’ve got to love and trust the people that are there for you. Even if you feel like you’re isolated or whatever, there are people that are there for you. So you have to put your trust in those people to feel like you’ve got the confidence to get help.

Participant, 12-to-15-years-old age group

At this stage of the journey, the app sends reminders for the scheduled to-do list tasks and allows users to add new activities, track their progress, and work toward creating habits. To minimize user effort, most activities can be modified and tracked with simple app interactions, such as tapping a checkbox to indicate a completed item, and are gamified using streaks and badges for increased engagement.

#### Stage 6: Maintaining Healthy Habits and Growth

Participants discussed that creating a positive structure around newly formed habits to maintain well-being constituted the final phase of the journey. Affirmation and recognition of the progress, usually within “the safety net” helped to prevent regression into earlier stages. Focusing on the enjoyment motivated young people to stay with the process at times of fallbacks. They described the “enjoyment line” as a

“line between depression/happiness“; that you can have enjoyment; that enjoyment can actually bring you up. So if there's that line, when you reach a point where you actually have enjoyment again; so enjoyment can bring you further up; and then more care and love can bring you up to the point of wellbeing and happiness...

Participant, 16-to-19-years-old age group

It’s probably movable. It’s not in a straight way. You’re kind of like ‘oh I’ve made so much progress on myself and I’ve come such a long way and I think that’s good’. And it just boosts you up a bit more.

Participant, 16-to-19-years-old age group

This stage incorporated an additional aspect of growth. Participants’ descriptions revealed that, having successfully taken the recovery journey, young people felt compelled to open up to others and offer advice, support, and help and that, at that time, personal experience could be used to normalize and empower others’ journeys to well-being.

This stage of the journey was supported in the app through (1) a sophisticated calendar tracking feature where users can monitor their progress and logs daily, weekly, or monthly; (2) gamification—achieving a new habit by completing a series of to-do items was rewarded by the *to-do* icon moving above the enjoyment line; and (3) inviting users to submit their personal recovery story of growth and their own niggles with other app users, thus closing the recovery cycle.

### The Niggle App

**Figure 7:**
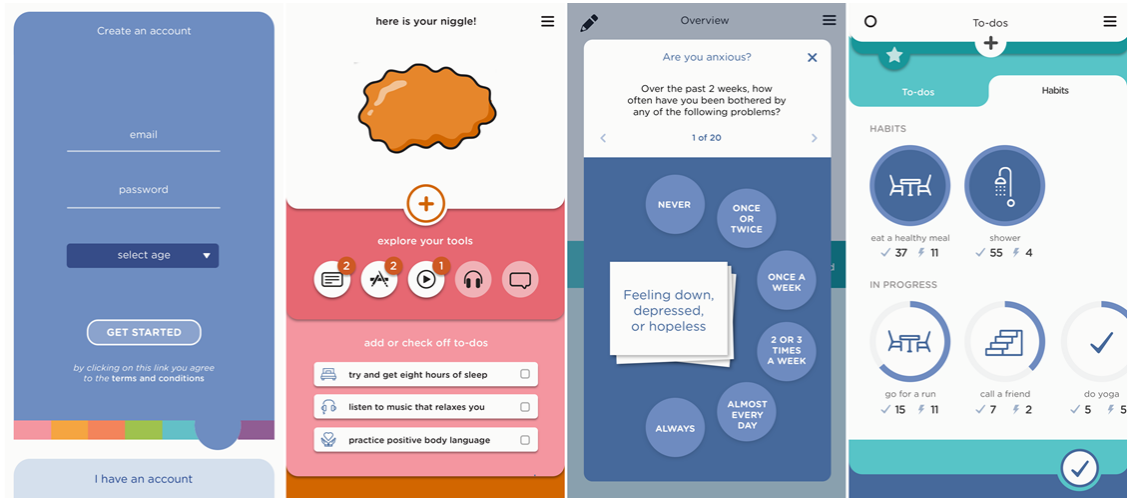
App features to increase engagement.

The app name was chosen by participants from the youngest age group (12-15 years) and was incorporated as a core concept of the app. Users are invited to reflect on their current physical and psychological well-being, identify their concerns or niggles, step back and reflect on their emotions, consider whether they need to discuss them with Kids Helpline, read about others’ similar experiences, learn from a wealth of carefully selected information and resources, plan to-do lists, take action, and track their progress. Upon successfully completing the recovery journey, they can share their story with peers. There are additional app features to increase the customizability, usability, and engagement of the app. For example, users can select from 7 color templates or use quizzes to assess their distress, resilience, and well-being (Figure 7). A notable positive aspect of the recovery journey involves being able to access a network of support from family, friends, and the community, which Niggle aimed to expand by creating an in-app community and including hotlink buttons to Kids Helpline as the first version of users’ “safety net.” Using the hotlink offers app users queue priority for receiving immediate support. Multiple additional features were conceptualized based on the workshops and will gradually be implemented in future app updates.

## Discussion

### General

This paper presents the theoretical development and concept design phases of Niggle—a smartphone app to assist young people on every step of their recovery journey to well-being. App quality was ensured by following principles outlined by the MARS [35].

We grounded our research in the experiences of young people through participatory research [38,40,41,50] and co-design [37] to develop a clear understanding of their recovery journey and to allow young people to have more control over the process. While thematic analysis was rigorously applied to interpret data [53] and emerging themes were commonly supported by participants, we acknowledge that variability may exist regarding experiences in view of age, gender, ethnicity, race, and class differences. The young people who participated generally described the recovery journey as a multistage process, and their group discussions helped define 6 distinct stages. There was strong consensus that transition through these stages was nonlinear, and each stage was characterized by multiple barriers and helpful strategies. Therefore, Niggle corresponds to the intervention-delivery model through its nonlinear and highly customizable design.

Journey mapping allowed us to explore the barriers that young people encounter in help seeking and how they differ at each stage. An in-depth understanding of these concerns can assist health care providers in better addressing a young person's needs. For example, while denial, stigma, fearfulness, and avoidance seem to be present throughout the journey, specific logistics such as time, transportation, costs, and the availability of services are pertinent to the more active *planning action* and *taking action* stages.

Applying visual research methods allowed us to increase our confidence of overcoming the potential linguistic barriers that our youngest participants may have experienced. Thus, we present the useful strategies and tools young people rely upon to overcome barriers and how they can be translated into co-designed digital intervention features to support recovery.

Recovery has been theorized as being both an outcome and a process, and is still being debated in the literature [54]. Supporters of recovery as a process argue that its conceptualization as restoration of capacities to the stage preceding illness is partial and flawed, and our findings support this view. We found that recovery consists of the complementary experiences of restoration from mental illness and advancement through the stages of well-being (identifying, processing, and taking action, which results in growth). The themes that we identified largely align with those in descriptive psychological models of behavior change such as the transtheoretical model (TTM) [55]. The first stages of the TTM reflect a process of precontemplation (no intention to seek help in the near future) and contemplation (considering a change some time in the future based on the potential positives and negatives of that change) similar to those defined by our participants as *recognizing that something is not right* and *labeling the problem*). The subsequent TTM stages include preparation (an intention to seek help in the immediate future), corresponding to our *processing the problem* and *planning action* stages; the fourth TTM stage action (or currently seeking help) is similar to the *taking action* stage of the well-being journey. The TTM includes the stages of maintenance (help seeking when needed in an attempt to avoid relapse of problems) and termination (when help seeking is no longer required), which in our participants’ discourse emerged as *maintaining health habits and growth*. Lastly, in line with our findings, the TTM also recognizes that relapse is a common and normal part of the change cycle and defines ‘recovery’ as a process, rather than a state, or outcome. 

Aligned with those of Prochaska et al [55], our findings, based on the participants’ discussions, confirm that the recovery journey could be nonlinear. We argue that there is not a single, predetermined solution for dealing with each stage. While motivational work might assist with contemplation in the TTM, it may also be applicable to all stages of the journey. Similarly, skills training does not need to be limited to the action stage, as it may be appropriate for building confidence during contemplation or preparation. Our results reflect the TTM’s applicability to young Australians in an era of unprecedented technological development, information overload, and shifting help-seeking pathways.

Consistent with previous findings [31], our findings also identified barriers such as stigma, embarrassment, poor mental health literacy, and a preference for self-reliance to youth help seeking, while facilitators included perceived positive past experiences, and social support.

### Limitations and Further Research

Despite efforts to capture diverse experiences, the participant sample was relatively homogenous. Most participants identified as female, urban residents, educated, White, living with parents or guardians, and single. None were employed full-time. We presume that young people from other backgrounds (such as Aboriginal and Torres Strait Islander, or culturally and linguistically diverse groups) would have provided a different account of recovery, mental health, self-care, and support safety nets. Yet, further work by our team, outside of the scope of the current project, is ongoing to confirm the relevancy of the stages reflected in the app design to a diverse group of young people, that includes young people from Indigenous backgrounds, young people with mental health diagnoses, and young people with diverse socioeconomic status.

The fact that male participants did not attend the workshop for the youngest age group (despite initially signing up), may reflect previous findings showing that the lowest rate of mental health help seeking is among young men, with only approximately 13% engaging with mental health services compared to 31% among young women [56,57]. This phenomenon has been linked to stigma (male role expectations and perceptions of appearing weak) [58], as well as low mental health literacy [31,56]. We hope that the Niggle app will be used to address some of these issues by offering confidential access to support and psychoeducation to all users.

Using preselected photos to elicit discussions on well-being may have biased participant discussions; however, we believe that this photographic prompt served to open deeper discussions. Future research might benefit from using verbal and visual data in tandem (ie, both photo elicitation and photo voice [59]) and by encouraging young people to produce their own images.

App quality and efficacy evaluation projects, including feedback sessions with young people and a series of Randomized Controlled Trials will be carried out to determine the efficacy and effectiveness of Niggle.

### Implications for Practice

This study contributes to the field of well-being, mental illness, recovery, eHealth, and mHealth for young people. It demonstrates the usefulness of research into the alignment of theoretical models to specific demographics for the development of theoretically-sound digital health technologies. The recovery journey described by our participants confirms the applicability of the TTM [55] to support behavior change and well-being amongst young people in the context of eHealth. Despite international health policies increasingly placing well-being at their center [60], no agreement exists as to what constitutes well-being. The definitions presented in this paper, grounded in participants’ complex personal accounts can assist with the development of policies and guidance for well-being and recovery interventions, regardless of the delivery method.

While there is a vast number of health apps targeting a range of users and issues, many lack theoretical grounding, quality, reliability, and applicability to key stakeholders. This paper offers an insight into health app development through in-depth phenomenological research, co-design, adherence to app quality principles, and the involvement of diverse expertise.

### Conclusion

We hope that Niggle will help young people access useful resources and support to increase their well-being. As of November 2019, version 1 of the app is freely available on Australian app stores. In the first year it was used by 12,823 individuals. Global release is planned once data privacy and security standards for international transfer and management have been carefully implemented.

## Appendix

Multimedia Appendix 1 Well-being workshops complete and numbered image set.

Multimedia Appendix 2 Topic guide.

## Abbreviations

MARS: Mobile App Rating Scale
mHealth: mobile health
TTM: transtheoretical model
