# Supplementary figures and images for: Development of the Niggle App for Supporting Young People on Their Dynamic Journey to Well-being: Co-design and Qualitative Research Study
Source: JMIR Mhealth Uhealth. 2021 Apr 20;9(4):e21085. doi: 10.2196/21085 (PMC8097523; doi:10.2196/21085)

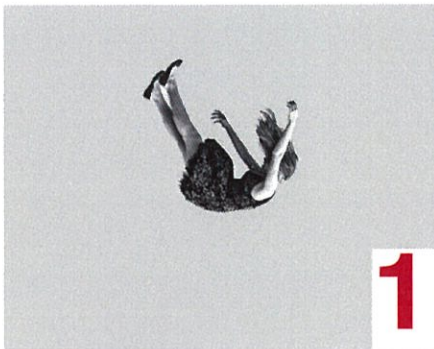

1

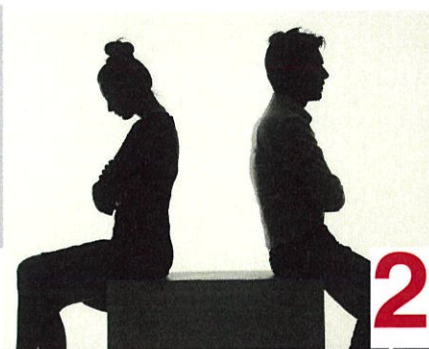

2

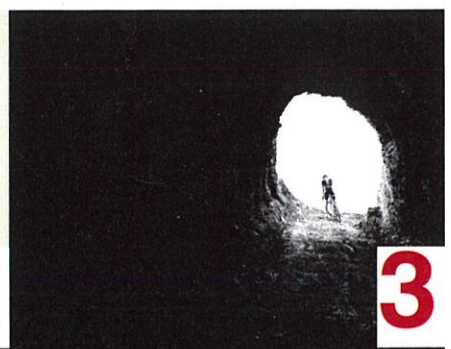

3

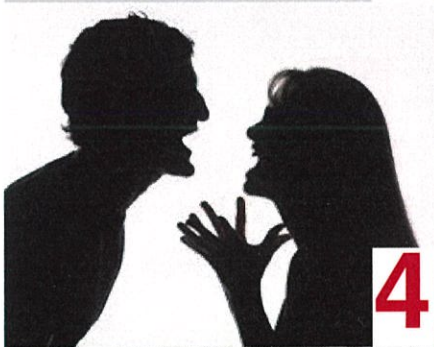

4

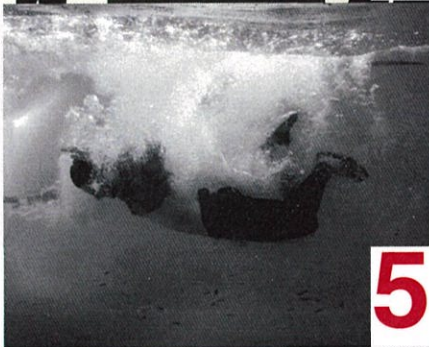

5

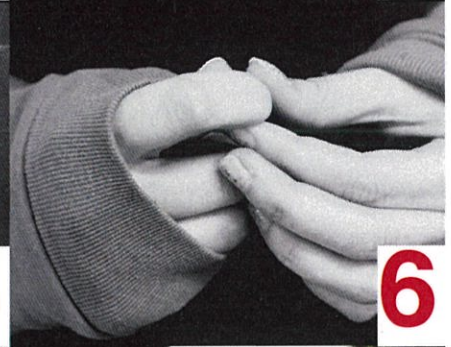

6

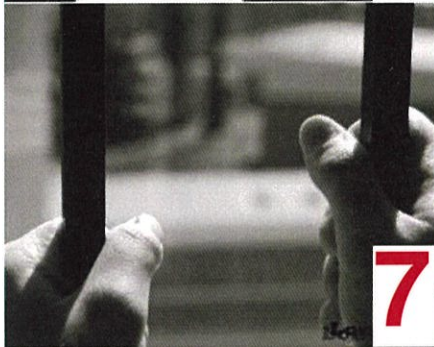

7

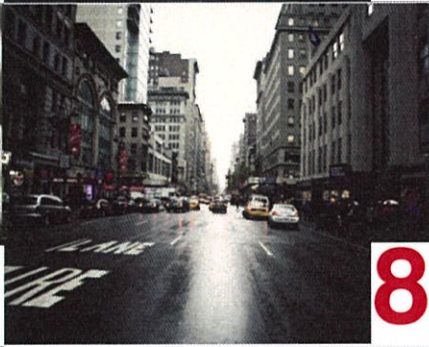

8

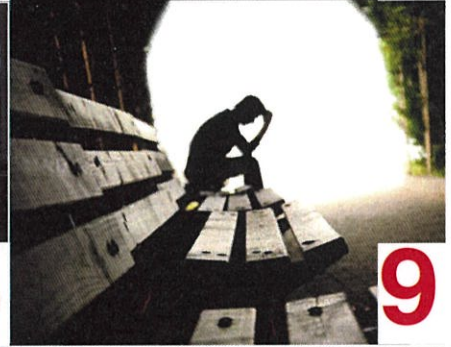

9

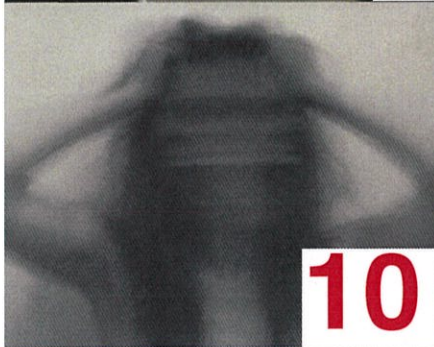

10

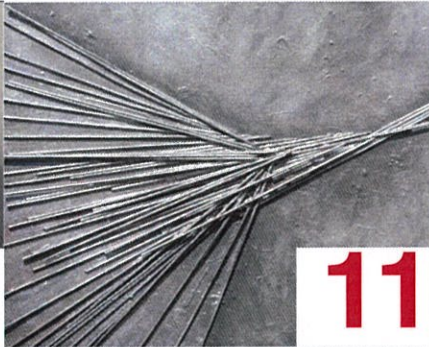

11

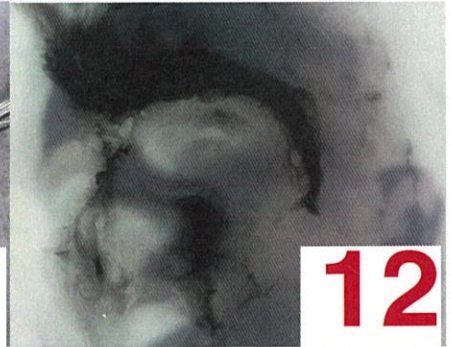

12

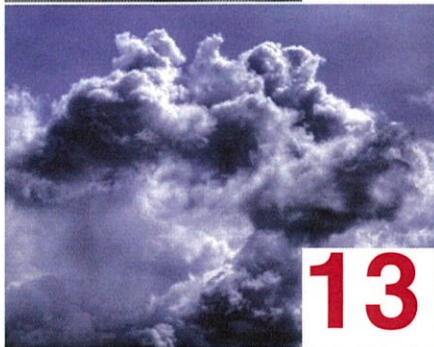

13

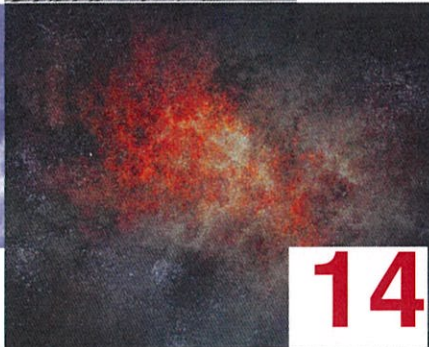

14

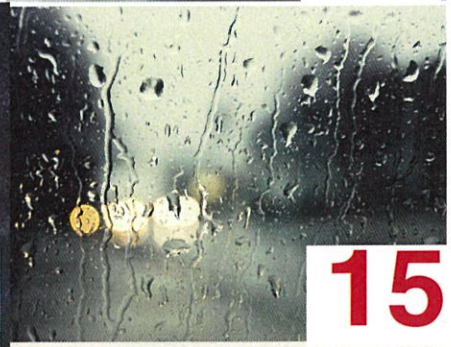

15

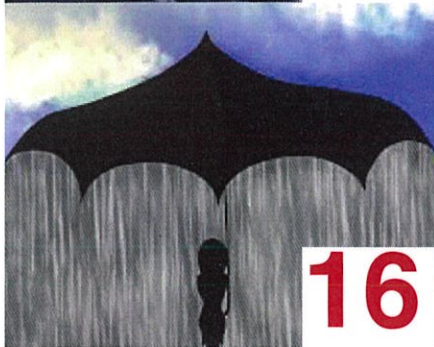

16

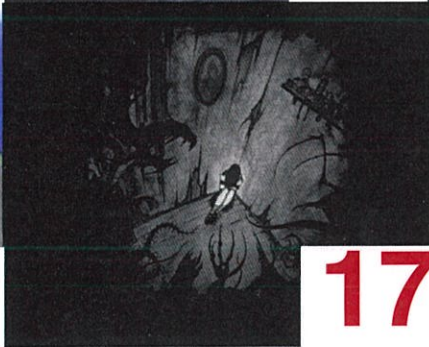

17

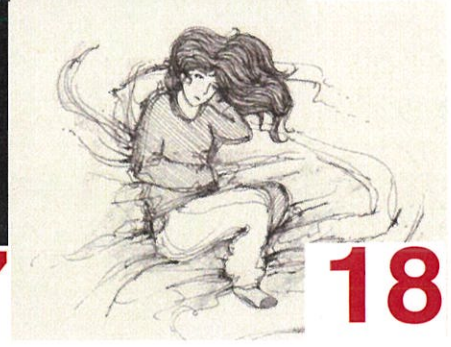

18

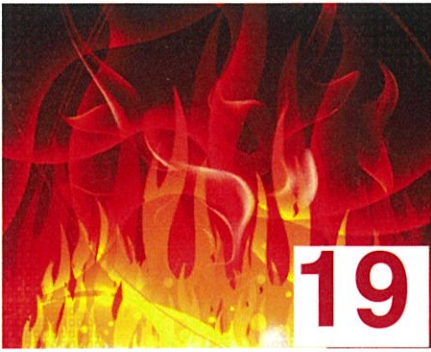

19

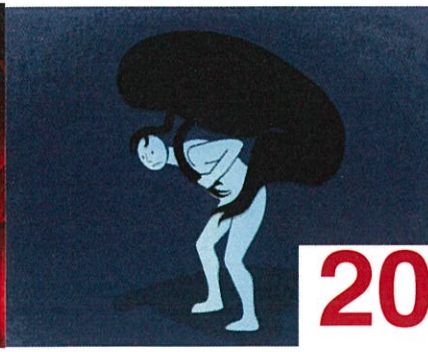

20

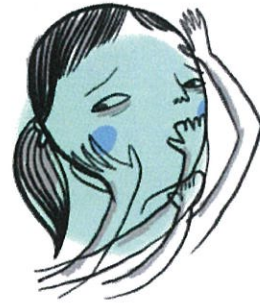

21

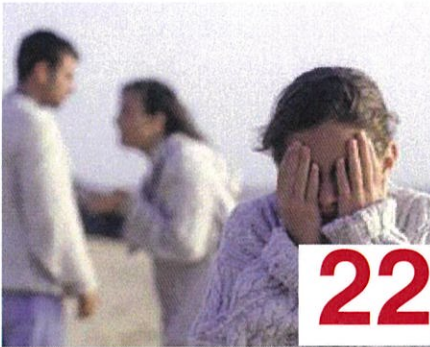

22

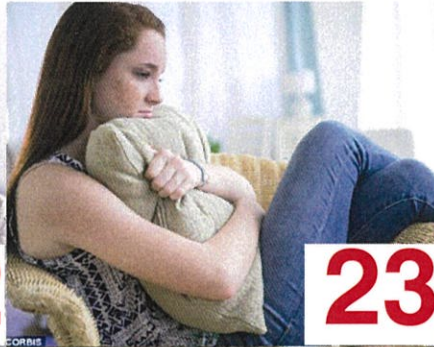

23

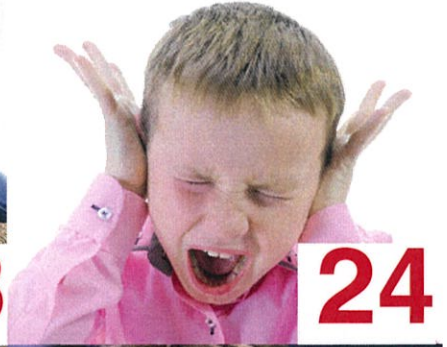

24

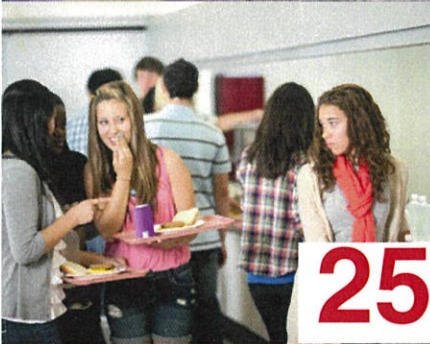

25

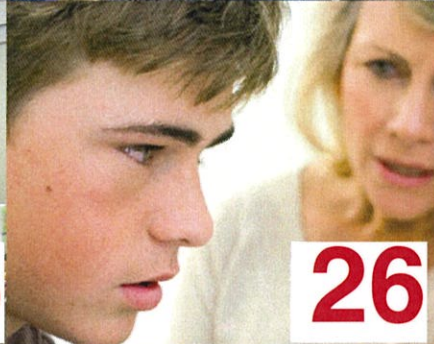

26

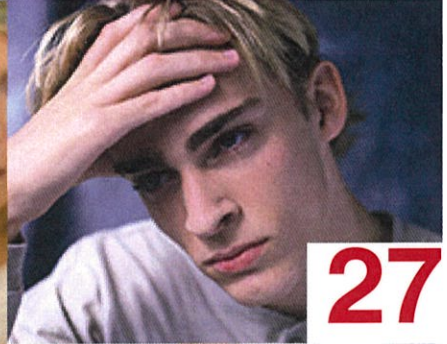

27

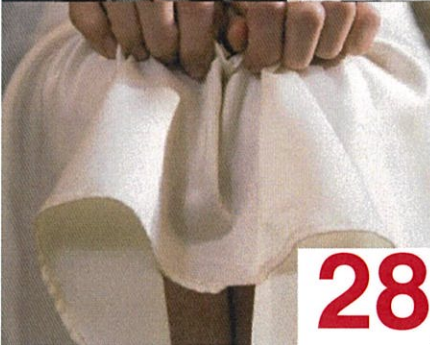

28

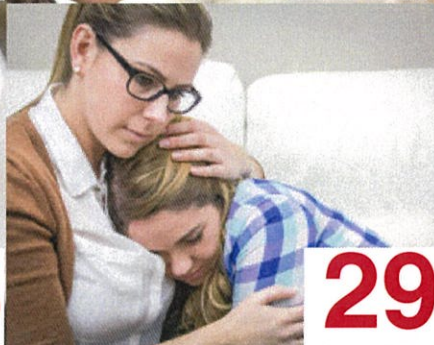

29

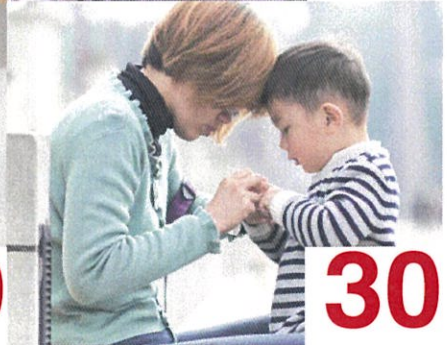

30

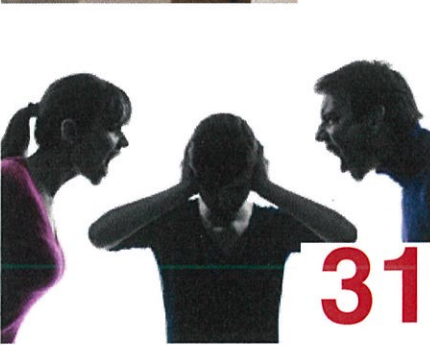

31

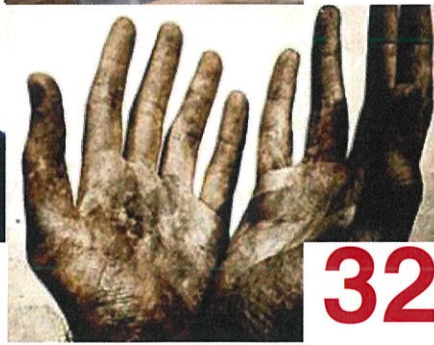

32

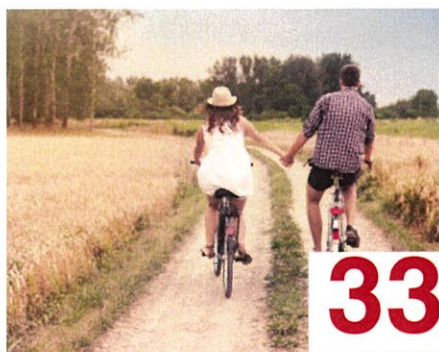

33

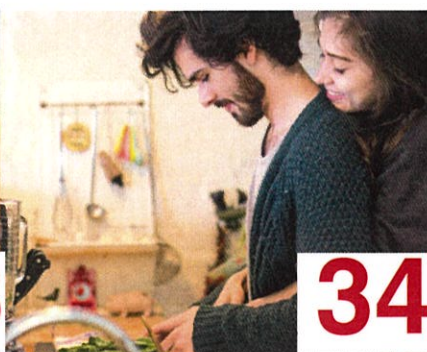

34

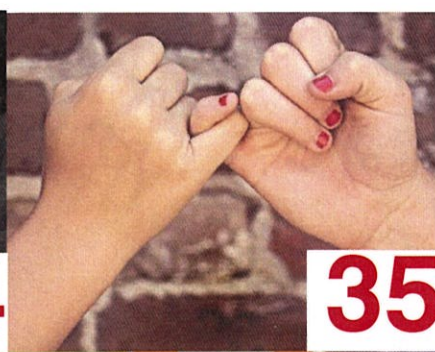

35

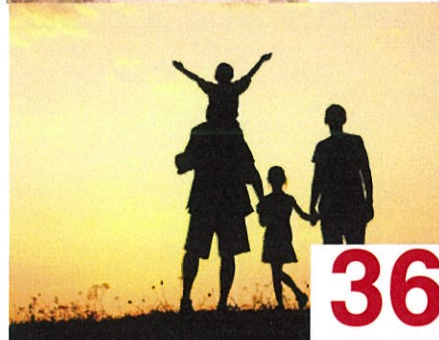

36

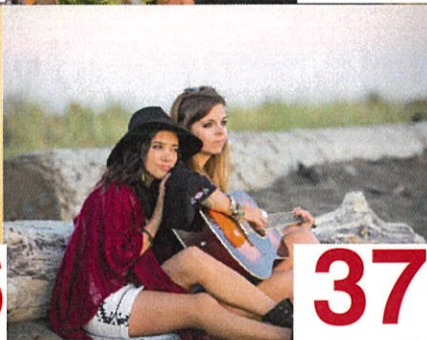

37

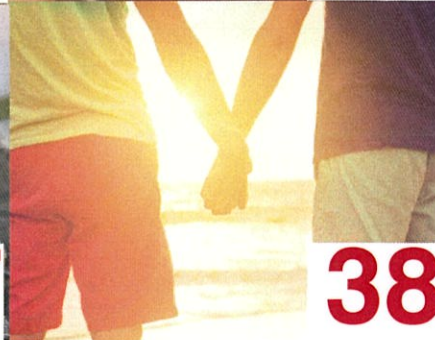

38

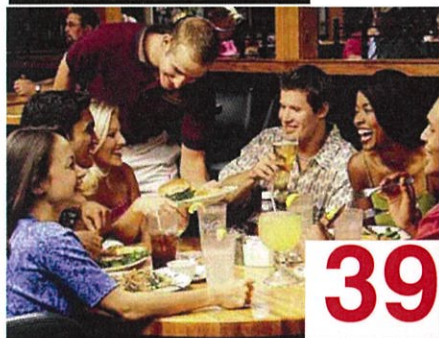

39

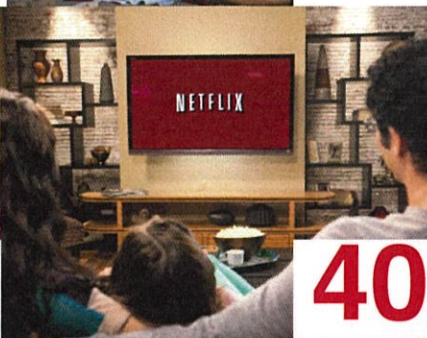

40

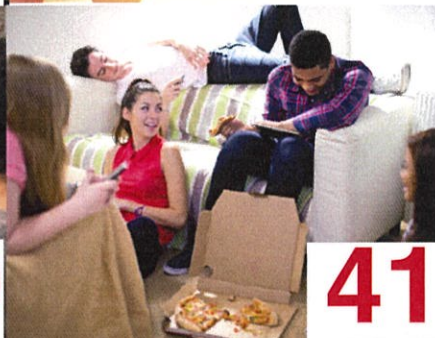

41

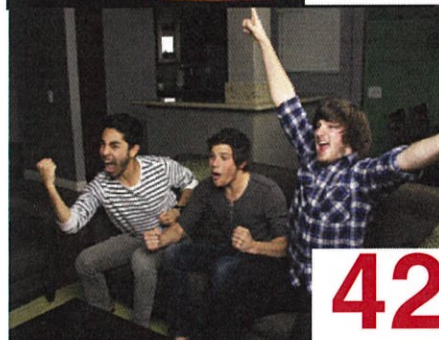

42

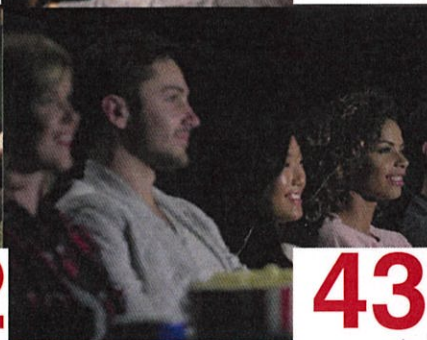

43

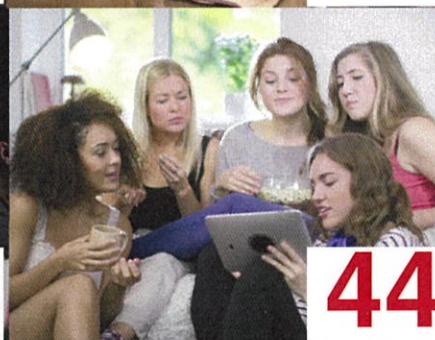

44

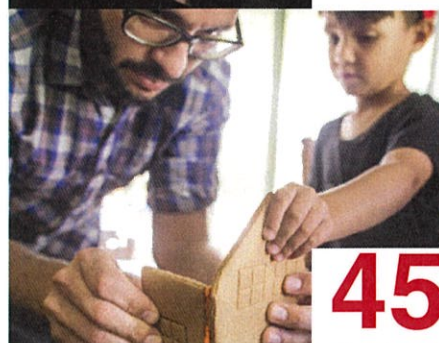

45

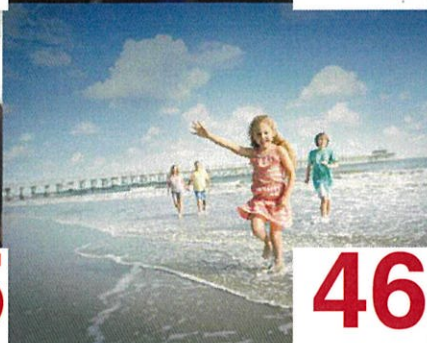

46

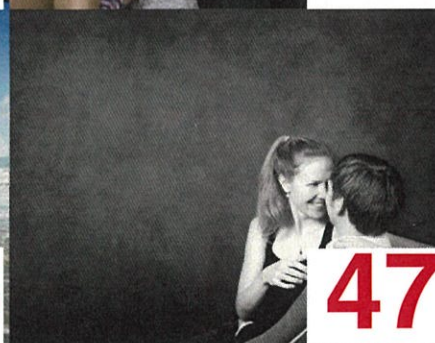

47

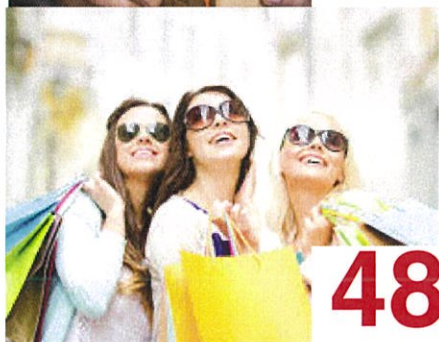

48

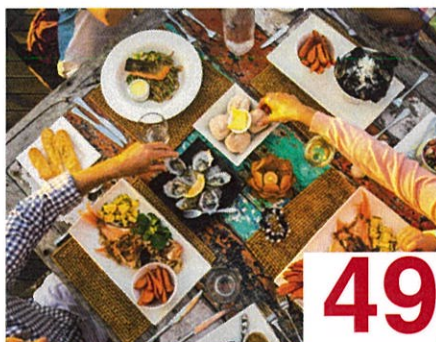

49

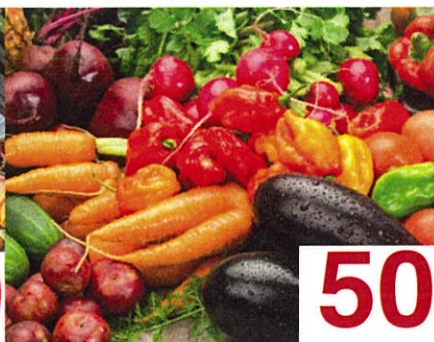

50

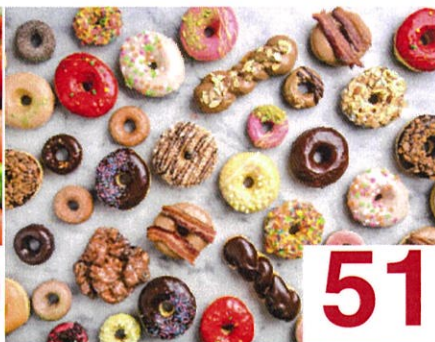

51

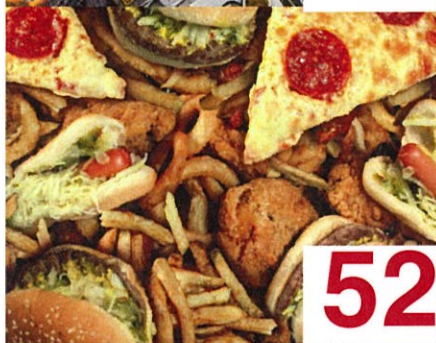

52

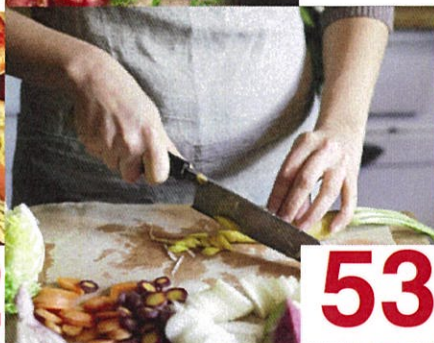

53

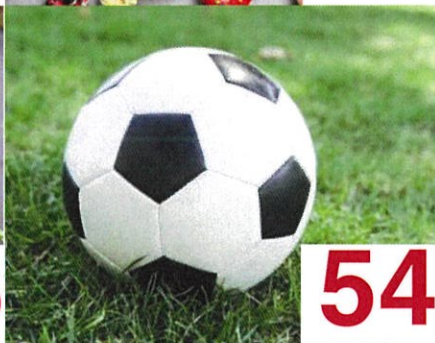

54

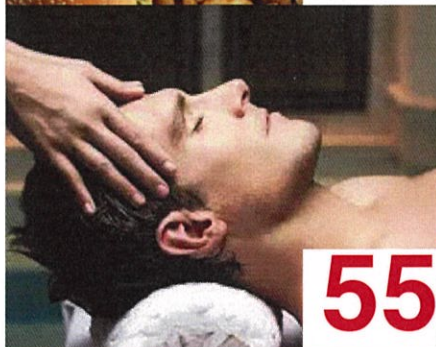

55

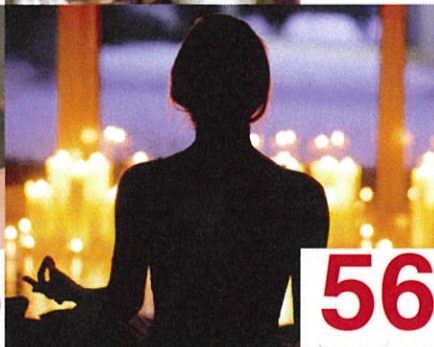

56

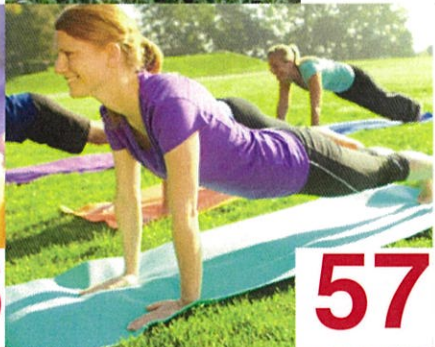

57

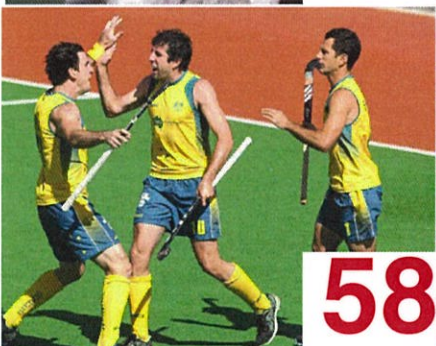

58

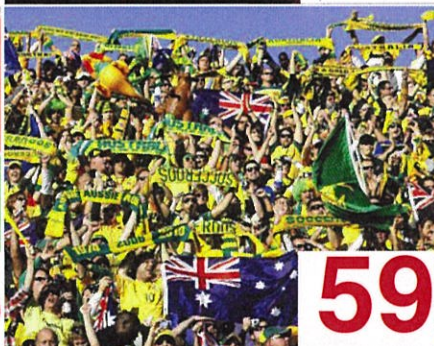

59

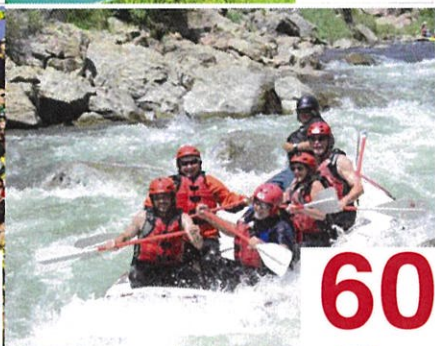

60

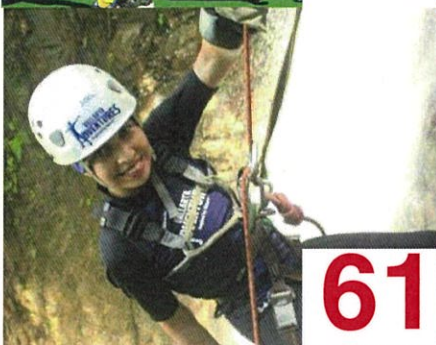

61

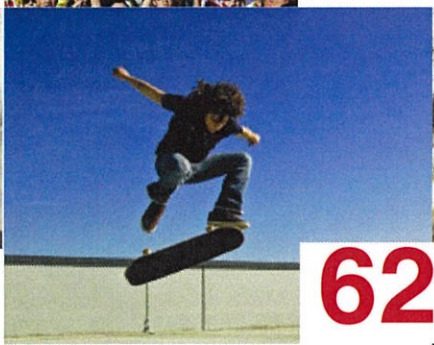

62

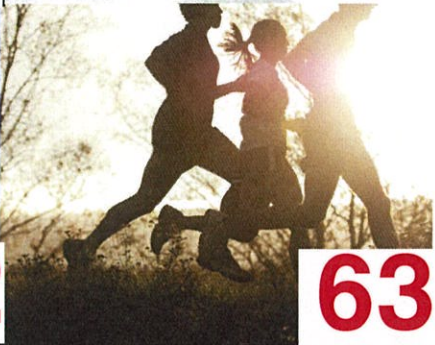

63

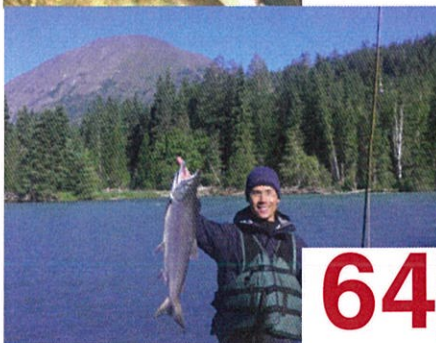

64

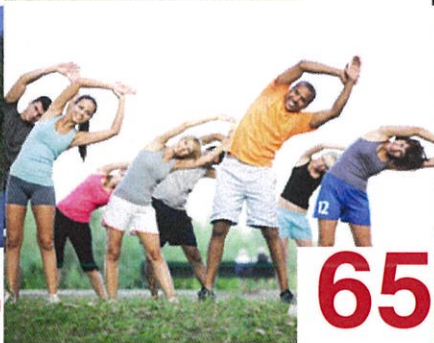

65

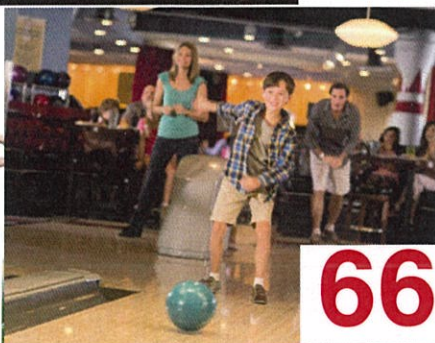

66

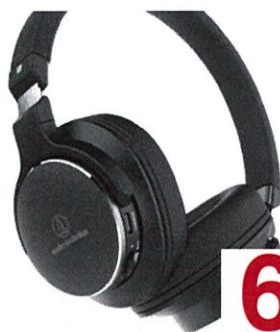

67

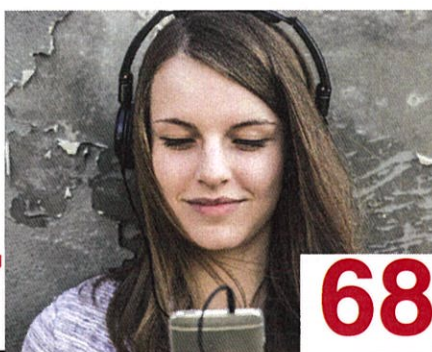

68

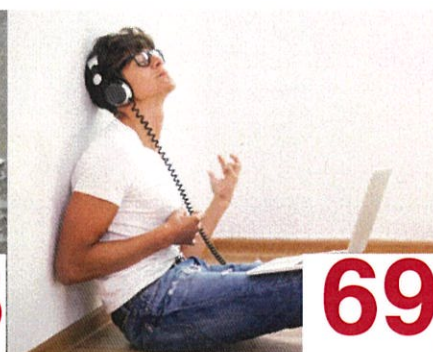

69

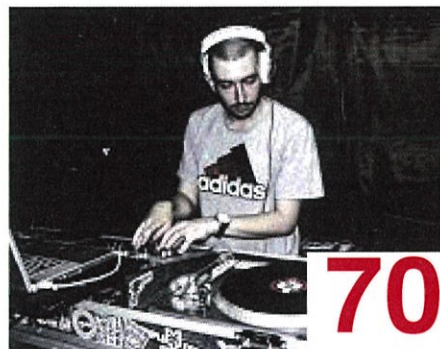

70

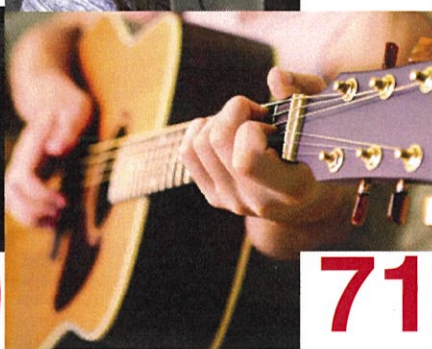

71

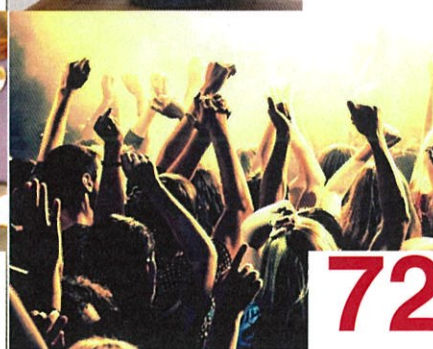

72

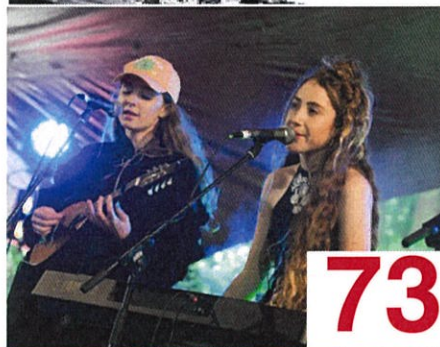

73

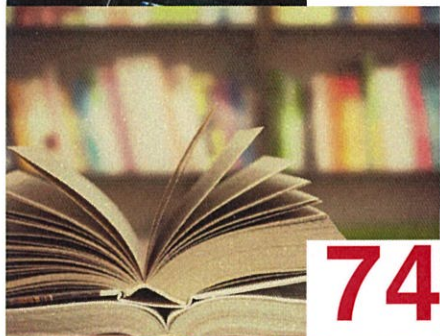

74

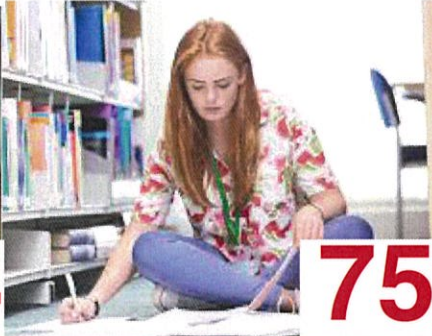

75

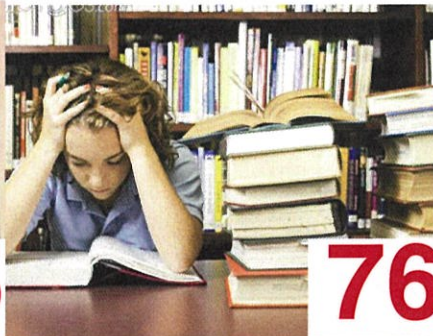

76

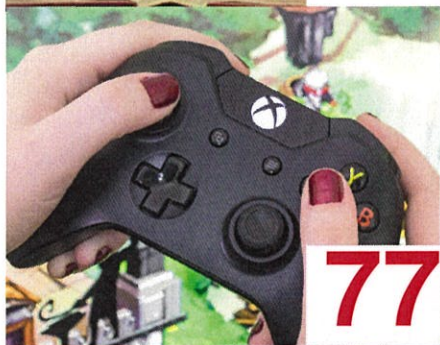

77

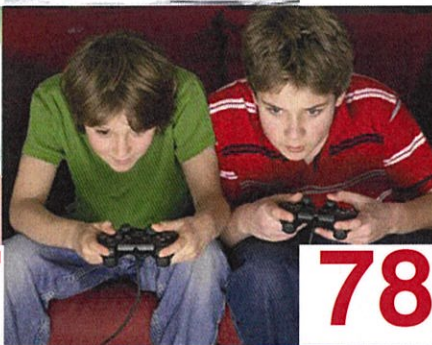

78

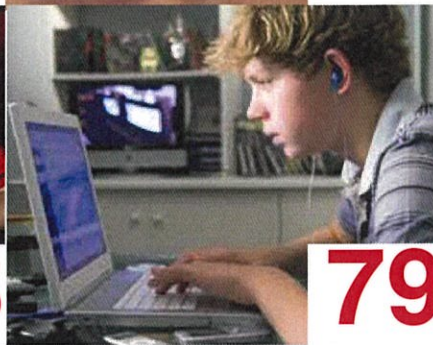

79

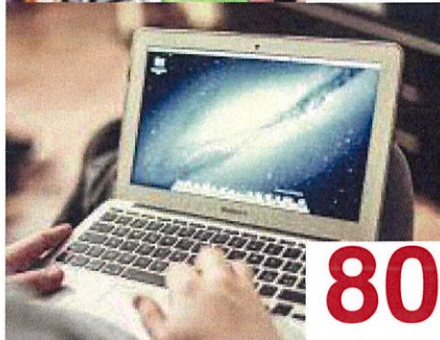

80

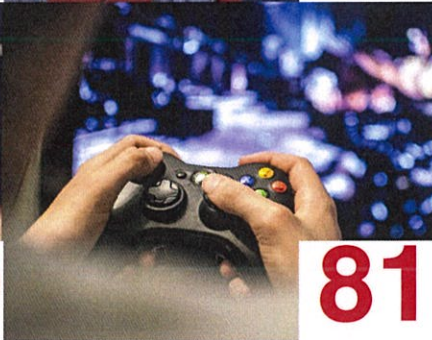

81

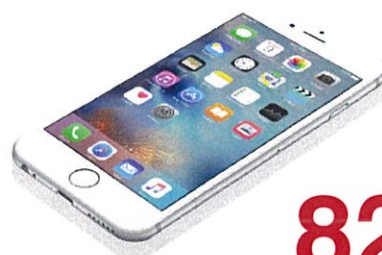

82

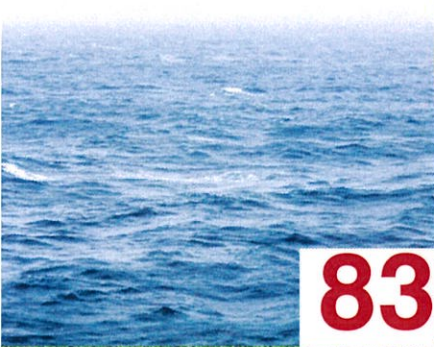

83

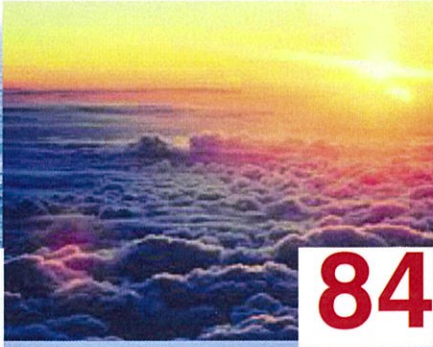

84

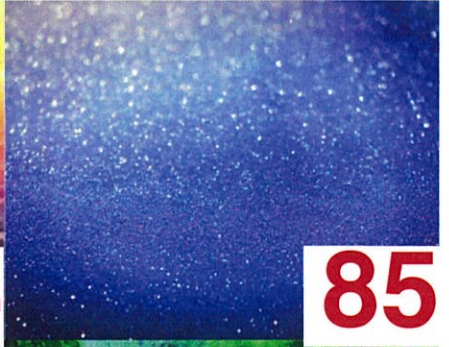

85

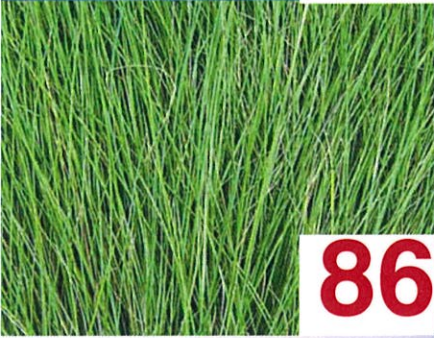

86

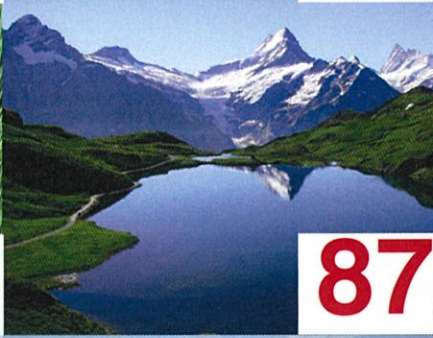

87

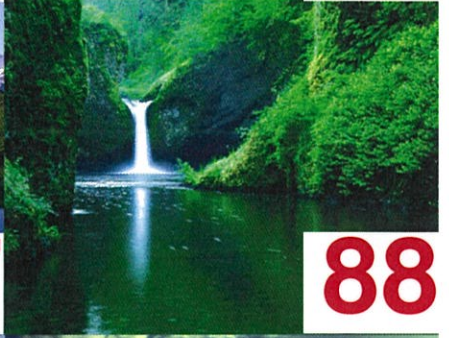

88

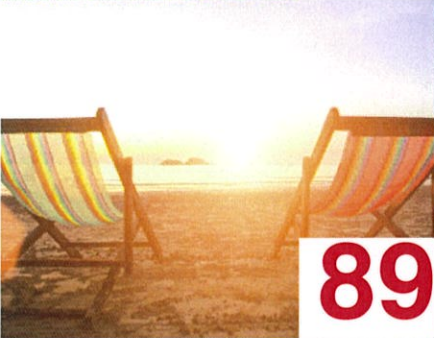

89

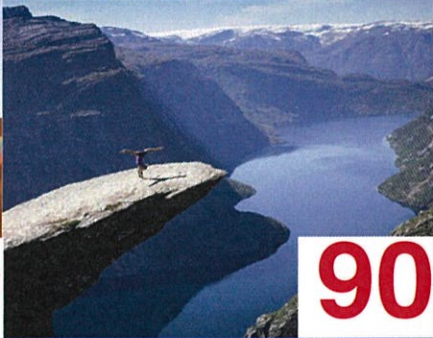

90

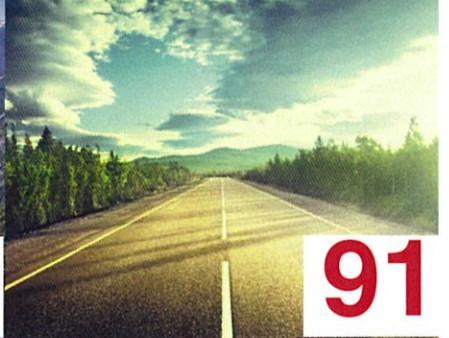

91

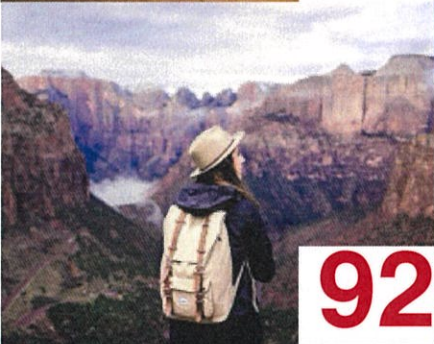

92

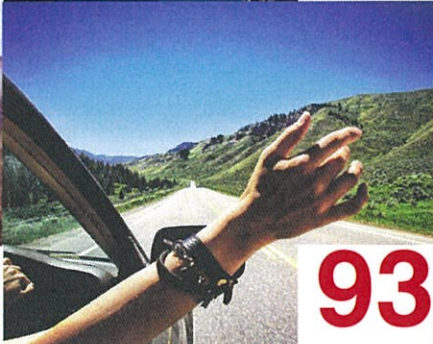

93

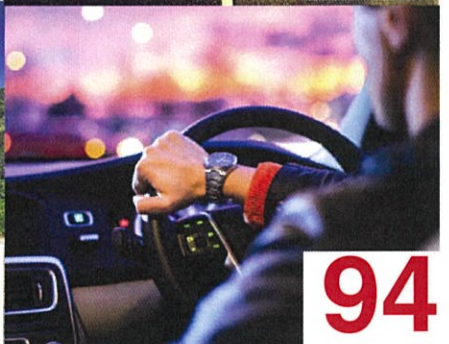

94

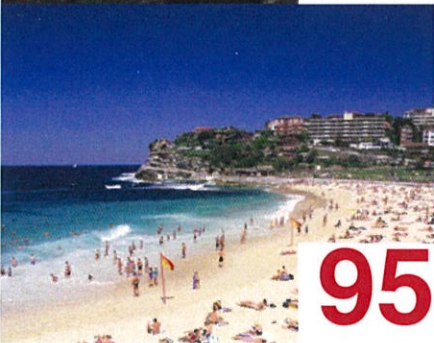

95

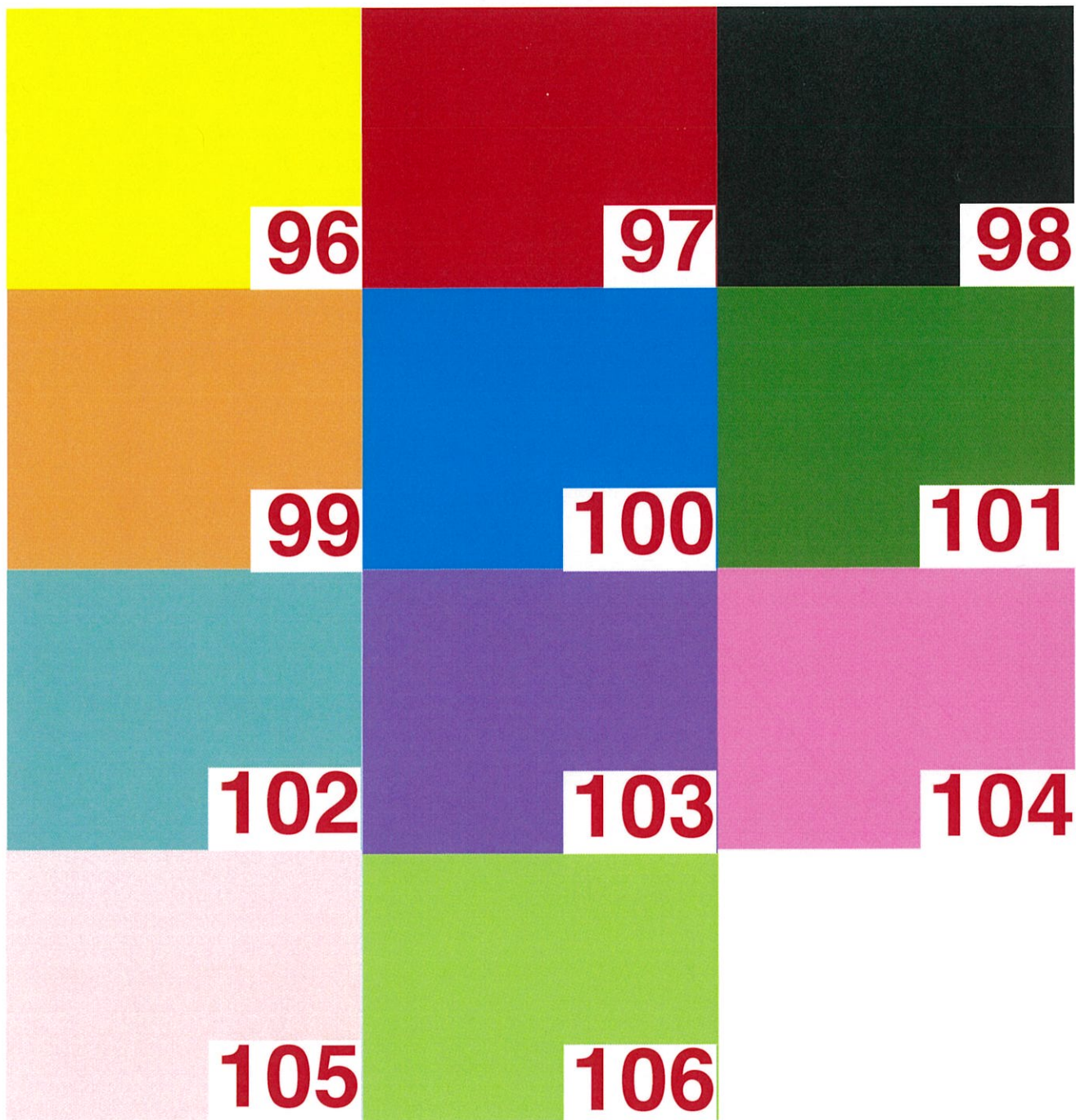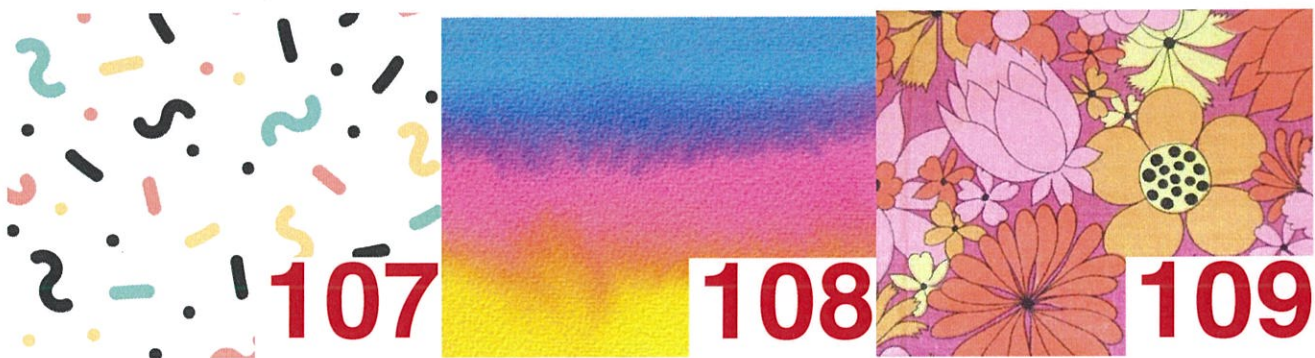

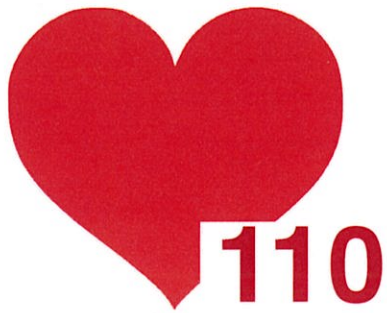

110

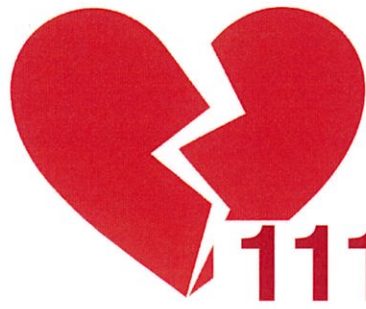

111

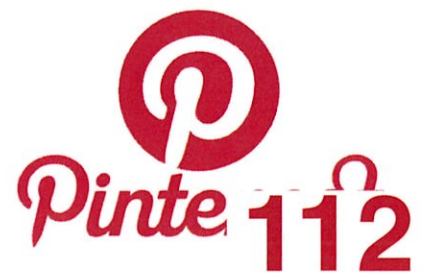

112

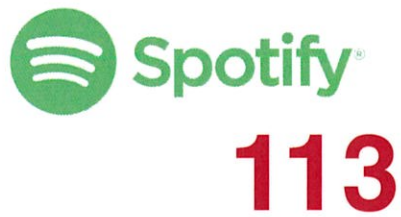

113

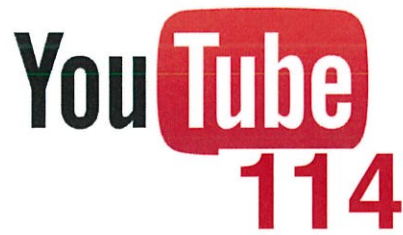

114

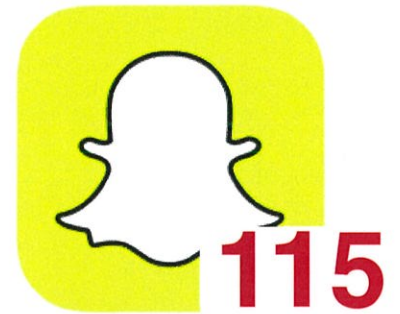

115

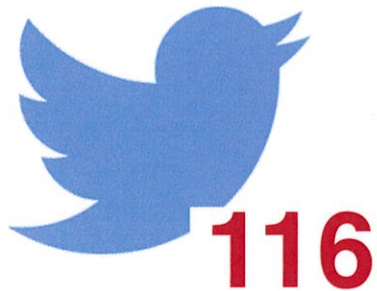

116

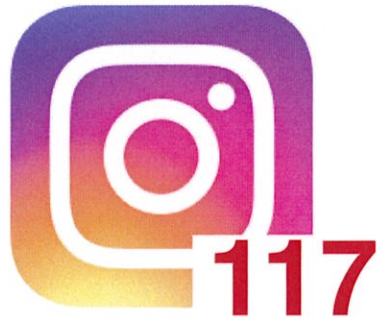

117

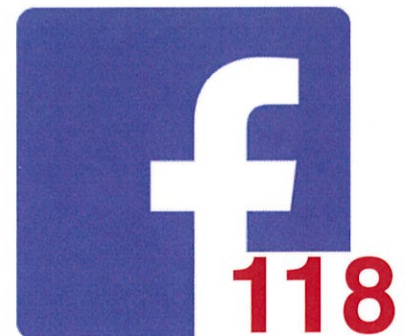

118

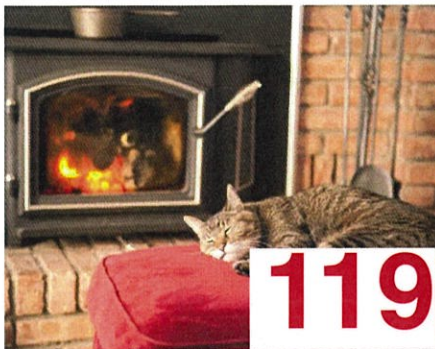

119

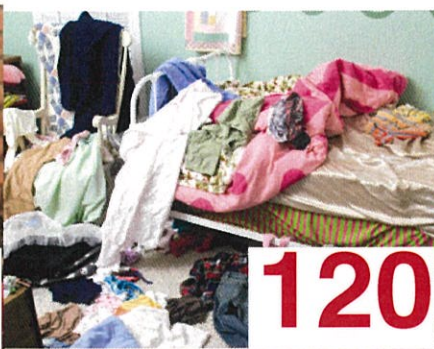

120

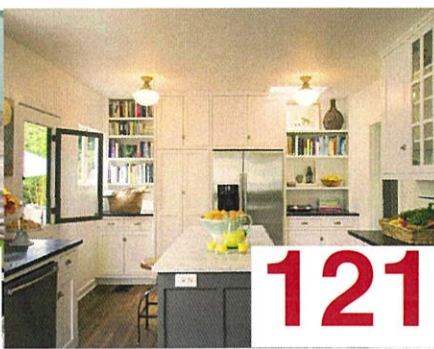

121

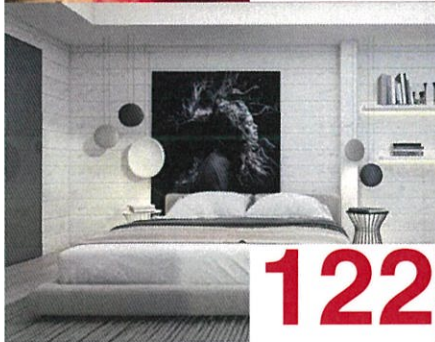

122

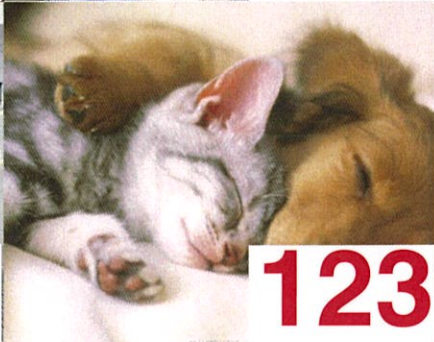

123

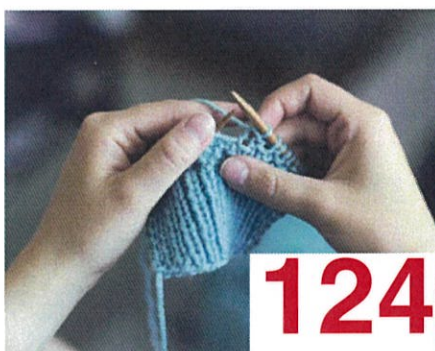

124

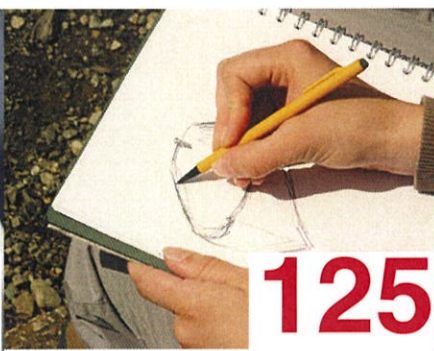

125

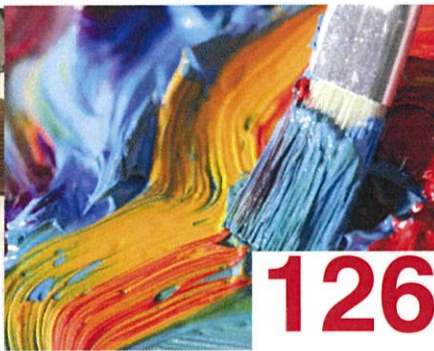

126

Supplement: Multimedia Appendix 1 [file mhealth_v9i4e21085_app1.pdf]
