# Supplementary material for: Development of the Niggle App for Supporting Young People on Their Dynamic Journey to Well-being: Co-design and Qualitative Research Study
Source: JMIR Mhealth Uhealth. 2021 Apr 20;9(4):e21085. doi: 10.2196/21085 (PMC8097523; doi:10.2196/21085)
Supplement: Multimedia Appendix 2 [file mhealth_v9i4e21085_app2.docx]

## Workshop 1 – wellbeing and journeys - general

**Activity 1: Icebreakers** drawing and animal associations

**Activity 2: Free associations of Wellbeing –** write list

**Activity 3: Free associations of Being un-well –** write list

**Activity 4: Wellbeing image collage –** image selection, sticking on paper, and writing annotations

**Activity 5: Being unwell collage –** image selection, gluing sticking on paper, and writing annotations

**Activity 6: Journey maps –** choose words signifying “being unwell” and “wellbeing” to you

what steps would you take to get from the point of “being unwell” to the point of wellbeing? Write or give us as much detail about each step as you can.

**Activity 7: Group journey discussion and synthesis -** If you feel comfortable doing so, let us talk about the journeys you have created.

**Activity 8:** Debrief

## Workshop 2 - use of technology for information, mental health and wellbeing

**Activity 1: Applicability of technology –** Please review your journey map**.** What technological functions would best address youth needs in progressing through the journey to wellbeing? (prompts: At what steps would you use technology for support? Are there any steps where you would prefer not to use technology?)

**Activity 2:** If money and resources were not an issue to design any type of mental health and wellbeing website/app/online media platform, what features would you find to be essential, useful, and engaging? (groups)

**Activity 3: Group discussion and synthesis –** presenting and discussing digital solutions

## Workshop 3 - explore design suggestions for the ToolKit

**Activity 1:** From the collated information from both groups in the previous Workshop– discuss in small groups the proposed features that you would find most engaging and why?

**Activity 2**: How would you suggest the differences and limitations could be overcome?

**Activity 3**: Final debrief
